# Rapid Mapping of Protein Interactions Using Tag‐Transfer Photocrosslinkers

**DOI:** 10.1002/anie.201809149

**Published:** 2018-11-21

**Authors:** Jim E. Horne, Martin Walko, Antonio N. Calabrese, Mark A. Levenstein, David J. Brockwell, Nikil Kapur, Andrew J. Wilson, Sheena E. Radford

**Affiliations:** ^1^ School of Molecular and Cellular Biology, Faculty of Biological Sciences University of Leeds Leeds LS2 9JT UK; ^2^ School of Chemistry University of Leeds Leeds LS2 9JT UK; ^3^ Astbury Centre for Structural Molecular Biology University of Leeds Leeds LS2 9JT UK; ^4^ School of Mechanical Engineering University of Leeds Leeds LS2 9JT UK

**Keywords:** chemical crosslinking, diazo compounds, mass spectrometry, photoaffinity labeling, protein–protein interactions

## Abstract

Analysing protein complexes by chemical crosslinking‐mass spectrometry (XL‐MS) is limited by the side‐chain reactivities and sizes of available crosslinkers, their slow reaction rates, and difficulties in crosslink enrichment, especially for rare, transient or dynamic complexes. Here we describe two new XL reagents that incorporate a methanethiosulfonate (MTS) group to label a reactive cysteine introduced into the bait protein, and a residue‐unbiased diazirine‐based photoactivatable XL group to trap its interacting partner(s). Reductive removal of the bait transfers a thiol‐containing fragment of the crosslinking reagent onto the target that can be alkylated and located by MS sequencing and exploited for enrichment, enabling the detection of low abundance crosslinks. Using these reagents and a bespoke UV LED irradiation platform, we show that maximum crosslinking yield is achieved within 10 seconds. The utility of this “tag and transfer” approach is demonstrated using a well‐defined peptide/protein regulatory interaction (BID_80‐102_/MCL‐1), and the dynamic interaction interface of a chaperone/substrate complex (Skp/OmpA).

Chemical crosslinking‐mass spectrometry (XL‐MS) is a powerful component of the structural biology toolkit.^1^ XL‐MS methods have been enhanced by new data analysis software,^2^ cleavable crosslinkers,^3^ strategies for crosslink enrichment,^4^ footprinting reagents,^5^ and structural modelling approaches.^6^ However, several challenges remain. For example, many XL‐MS reagents have limited chemical reactivities (e.g. succinimide ester‐based reagents)^7^ restricting the residues for which information can be obtained. Secondly, the low‐abundance of crosslinked products often necessitates enrichment prior to MS.^8^ Finally, crosslinked peptide identification is difficult due to spectral complexity, poor fragmentation efficiency, and the increased search space associated with the large numbers of peptide combinations.^2^ Bulky crosslinking reagents may also perturb native interactions or create new aberrant interactions.^9^ Furthermore, many biological processes, for example, protein folding, binding and conformational changes, occur on short timescales. Consequently, crosslinks detected for a dynamic/non‐equilibrium system report the average of multiple states or are dominated by the longest‐lived state of all those populated. Photocrosslinkers such as diazirines can address this challenge and have been used previously to tag Cys residues in peptides.^10^ Diazirine‐generated carbenes react with proteins in ns,^11^ yet long irradiation times (minutes to hours10b, 12) are often required to generate acceptable crosslink yields due to the use of low intensity lamps.

Here we exploit a “tag and transfer” approach^13^ to develop two new XL‐MS reagents and a workflow that enables crosslink identification for both well‐defined and dynamic protein‐protein interactions (PPIs). These heterobifunctional reagents comprise a methanethiosulfonate (MTS) group for specific attachment onto a single Cys residue introduced into the “bait” protein, creating a cleavable disulfide bond within the linker arm, and a diazirine that crosslinks to a “target” protein (Figure 1 a,b, Figure S1,S2). Commercially available MTS‐benzophenone‐biotin tags have been used in photoinduced‐crosslinking (PI‐XL), but these bulky tags (752 Da) may perturb PPI interfaces.^14^ In the reagents described here, reduction of the disulfide bond‐containing linker arm between the crosslinked proteins leaves a thiol tag on the target (87 or 204 Da in size) (Figure 1 b, Figure S3). The modified residue can then be alkylated (e.g. with iodoacetamide [IAA]) and localised using MS protocols for identifying post‐translational modifications (PTMs),^2^ or enriched before MS. We also describe the construction of a 365 nm UV LED irradiation platform (Figure 1 c, Figure S4a,b) which enables crosslinking reactions to be completed in just 10 seconds with marginal heating. We exemplify this methodology by mapping two PPIs, each with a different binding mode: a complex between BID_80–102_ and MCL‐1 (*K*_D_=50±20 nm),^15^ where the tight binding affinity is mediated by several key residues15a, 16 and the dynamic chaperone/substrate complex Skp/OmpA (*K*_D_=22±16 nm),^17^ in which the binding interface involves many rapidly interconverting interactions.^17^, ^18^

**Figure 1:**
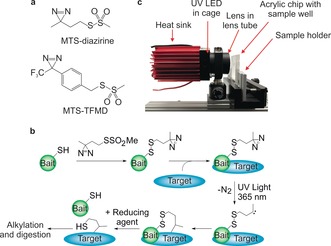
a) Structures of MTS‐diazirine and MTS‐TFMD. b) Crosslinking workflow schematic: A Cys‐containing bait protein is conjugated with the reagent (here MTS‐diazirine). After adding the target protein, the sample is irradiated with 365 nm UV light, revealing a carbene that reacts with the target. Reductant is added, leaving a sulfhydryl tag on the target at the interaction site. c) Image of the UV LED lamp and custom‐built acrylic chip comprising a 33 μL sample well. See also Figure S4a,b.

MTS‐diazirine and MTS‐trifluoromethyl phenyl diazirine (MTS‐TFMD) (Figure 1 a, Figure S1) were chosen as photoactivatable groups due to the superior performance of diazirines in comparative PI‐XL studies,12c, 19 their small size, and rapid, indiscriminate reactivity (Figure S2).^9^, ^11^ Both diazirine‐ and TFMD‐containing crosslinkers were synthesised as the photochemistry of TFMD leads to higher crosslinking efficiency, but it is bulkier.^20^ In the bait, a unique solvent exposed Cys for crosslinker conjugation is introduced by mutagenesis or synthesis. In proteins/peptides which lack Cys, or contain buried Cys, this is straightforward. When a solvent exposed Cys is already present in the bait this can be exploited, or substituted with Ala or Ser, and a new Cys introduced in a location of interest. Knowing the location of the Cys on one partner in the PPI reduces the MS/MS search space from *n*_tot_^2^ to *n*_target_ (where *n*_tot_ is the number of crosslinkable residues in the bait and target—for diazirines, this is every residue in the proteins—and *n*_target_ is the number of residues in the target). Here, we introduce a unique Cys by mutagenesis into the *E. coli* outer membrane protein OmpA, while for the BID_80–102_ peptide, which comprises the binding domain of the pro‐apoptotic protein BID, Cys was introduced via solid‐phase peptide synthesis. Importantly, the reactivity of the photoactivated diazirine places no restrictions on the amino acids in the target protein that can be detected once a complex is formed.

To enhance diazirine photoactivation efficiency we constructed a 365 nm LED lamp (Figure 1 c, Figure S4a,b) with a nominal flux density of 15 W cm^−2^. We designed devices to irradiate samples in 0.2 mL to 1.5 mL tubes, or in acrylic chips^21^ with 33 μL sample wells (Figure 1 c, Figure S5). Using this LED platform, maximal XL yields were achieved in 10 seconds, with an associated heating of <2 °C (Figure 2 a,b, Figure S4c). By contrast, a 6 W Hg‐Xe lamp achieved maximal yields after ca. 20 minutes with heating of 10 °C (Figure 2 a,b, Figure S4c), consistent with previous reports.10b, 12a,12b The UV LED design thus improves the reaction rate 130‐fold, and decreases heating 6‐fold.

**Figure 2:**
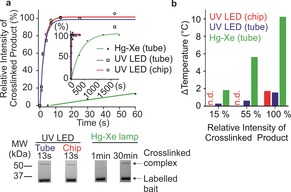
a) Densitometry quantification of the appearance of OmpA(W7C)[MTS‐diazirine] crosslinked to Skp vs. irradiation time. Inset: expanded time axis to show full time course using the Hg‐Xe lamp. Lines are exponential fits to the data. Relative intensity of crosslinked product [%] was calculated as the intensity of the crosslinked product band on an SDS‐PAGE gel at time *t* divided by the intensity at *t*_final_, assuming the maximal yield is achieved when the graph plateaus (see example SDS‐PAGE of the XL reaction (bottom) and Figure S4c). b) Sample heating at irradiation times required to reach 15, 55 and 100 % maximal crosslinked product for the Hg‐Xe and UV LED lamps. n.d.=not determined.

The human apoptotic regulatory pair BID/MCL‐1 is a target for cancer drug discovery.^22^ The BH3 binding domain of BID (BID_80–102_) adopts a helix on binding to a surface groove on MCL‐1 (Figure 3 a).15b, 16 Five single Cys variants of BID_80–102_ (Figure 3 a) were synthesised (see Supporting Information), each with a unique Cys residue in a different position. Subsequently, the peptides were tagged with MTS‐diazirine or MTS‐TFMD. The modified peptides bound MCL‐1 with high affinity, although those with the tagged residues in the centre of BID_80–102_ (I86C and V93C) had a reduced EC_50_ (Figure 3 b, Table S1). Crosslinking of the BID_80–102_ peptides with MCL‐1 was achieved by UV LED irradiation. Crosslinked complexes were detected by SDS‐PAGE (Figure 3 c). The absolute crosslinking efficiencies varied from 9–40 % for MTS‐diazirine, with reduced efficiency for lower affinity variants (Table S1). Absolute crosslinking efficiencies were higher for the MTS‐TFMD tagged peptides (26–53 %) (Table S1), as expected given the greater yield of TFMD‐derived carbenes.^20^ Gel bands of the crosslinked complexes were excised and the linker arm cleaved by reduction of the disulfide (Figure 1 b) releasing MCL‐1 bearing the transferred thiol‐containing tag at the interaction sites. The free thiol in each tag was capped by alkylation with IAA and the protein digested in‐gel with trypsin (see Supporting Information). Peptides modified with the transferred tag were identified by LC‐MS/MS. This allowed BID_80–102_ to be mapped into the MCL‐1 binding groove unambiguously, in agreement with the known “bind‐and‐fold” interaction^23^ and NMR/X‐ray structures of the complex (Figure 3 d, Tables S2 and S3, Figures S6–S8).15b, 16 Both crosslinkers gave similar results (Figure S6), demonstrating that the bulkier TFMD‐diazirine can be used even when sidechain interdigitation drives association. Crosslinked positions on MCL‐1 were quantified by MS/MS from each Cys residue introduced into BID_80–102_ labelled with MTS‐diazirine or MTS‐TFMD. This revealed that crosslinking yields were greater when the Cα–Cα Euclidian distance of the residues in the complex was <10 Å, but crosslinks could be detected for distances up to 15 Å (Figures S9,S10).

**Figure 3:**
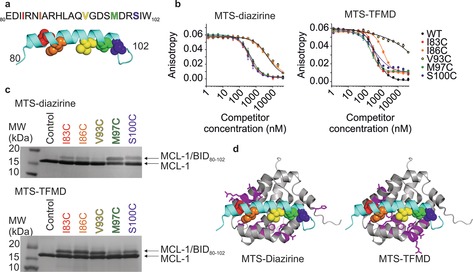
a) Sequence of WT BID_80–102_. Five Cys variants of BID_80‐102_ were labelled with MTS‐diazirine or MTS‐TFMD. Cys‐substituted amino acids are shown as coloured spheres in the peptide structure and in the same colour in the sequence above. b) Inhibitory potency (EC_50_) of BID_80–102_ labelled with MTS‐diazirine (left) or MTS‐TFMD (right) to MCL‐1 measured by fluorescence anisotropy (EC_50_ values are shown in Table S1). (c) SDS‐PAGE of BID_80–102_ labelled with MTS‐diazirine (top) or MTS‐TFMD (bottom) crosslinked to MCL‐1. d) Residues of MCL‐1 (magenta on a grey ribbon) that crosslinked to at least one BID_80–102_ peptide labelled with MTS‐diazirine (left) or MTS‐TFMD (right). BID_80–102_ is shown in cyan and residues substituted as Cys are coloured as in (a) (PDB ID: 2KBW^16^). See also Figures S6–S8, Tables S2, S3.

We next tested the ability of our workflow and tag transfer reagents to study a protein complex stabilised by transient interactions. Chaperone‐client binding often involves a dynamic interaction of chaperones with an unfolded/partially folded client protein to aid folding or prevent aggregation.^24^ These interfaces are challenging to map using crosslinking since the interactions can be dynamic and diffuse. Here, we used the interaction between the periplasmic chaperone Skp from *E. coli*, and a β‐barrel outer membrane protein substrate, OmpA, as a model for this type of PPI.^25^ Skp, a homotrimer, has a jellyfish‐like structure resulting in a cage in which substrates are sequestered (Figure 4 a).25b, 26 Skp binds its OMP substrates with nm affinity,^17^ similar to that of the well‐defined BID_80‐102_/MCL‐1 complex.^15^ However, Skp‐bound OmpA is in a “fluid‐globule” state that forms many weak and transient interactions in the Skp cage.^17^, ^18^

**Figure 4:**
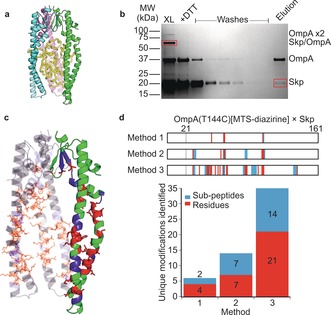
a) Model of the Skp/OmpA complex. Trimeric Skp (PDB ID: 1U2M^26^) is shown in cyan, pink and green. Collapsed OmpA (yellow) is shown in the Skp cavity.25b b) Non‐reducing SDS‐PAGE to show enrichment of crosslinked Skp/OmpA. The crosslinked Skp/OmpA(T144C)[MTS‐diazirine] complex (lane 1, XL) was cleaved with DTT. The constituent proteins contain free thiols so can be bound to thiopropyl Sepharose 6B. Unbound material was removed by washing and the captured material eluted by adding DTT. c) Structure of Skp with crosslinked residues from a pooled dataset of OmpA(T144C)[MTS‐diazirine] (Figure S13, Table S4). One monomer in the Skp trimer is highlighted. In c and d, red represents residue‐level information and blue represents crosslinks localised within a 2 or more residue region. d) Crosslinked residues identified by the enrichment methods shown in Figure 5 for OmpA(T144C)[MTS‐diazirine]. The lower plot shows the total number of unique sites identified by the three enrichment strategies shown in Figure 5. Representative mass spectra of modified Skp peptides are shown in Figures S15,S16 and a list of crosslinked peptides is in Tables S4,S5.

Two single Cys variants of OmpA were generated, W7C and T144C, located in the β‐barrel and a surface exposed loop in folded OmpA, respectively.^18^ MTS‐diazirine or MTS‐TFMD conjugation of both variants was efficient (Figure S11), and did not prevent folding (Figure S12). The Skp/OmpA complex was assembled by dilution of urea‐denatured OmpA into a Skp‐containing solution, and PI‐XL was then performed. The crosslinked Skp/OmpA complex was detected by non‐reducing SDS‐PAGE as a single band with an apparent mass of approximately 70 kDa (Figure 4 b). Four non‐overlapping positions from OmpA(T144C) to residues within Skp's “cage” (Figure 4 d (top); Figure S13) could be identified by in‐gel digestion of the crosslinked proteins followed by LC‐MS/MS (Figure 5, *Method 1*). This result was surprising since it is inconsistent with previous studies which have shown that OmpA tumbles dynamically on a sub‐ms timescale within Skp.^18^ We reasoned that the lack of modified sites resulted from the relatively low abundance of these peptides in the Skp‐OmpA complex, rather than reflecting a specific interaction surface involving these four sites. Given that each Skp trimer binds a single OmpA, and each OmpA contains only a single crosslinker, then only one modified peptide would result from each 70 kDa Skp/OmpA complex.

**Figure 5:**
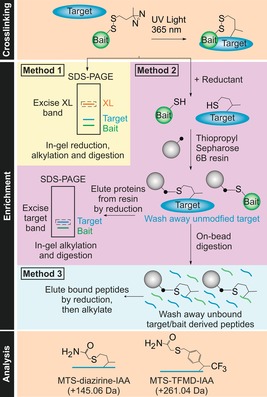
Enrichment and analysis strategies for MTS‐diazirine and MTS‐TFMD. Irradiation at 365 nm results in crosslinking of bait and target proteins (n.b. low reaction efficiencies mean that uncrosslinked material will remain). Enrichment can be performed using one of three methods (see the text). MS analysis of the peptides is then performed, and the data searched to identify the peptides/residues modified with the crosslinking reagent (with the free thiol capped by reaction with IAA).

To test this conclusion we developed a protocol to enrich chemically modified Skp, removing background peptides arising from OmpA and unmodified Skp (Figure 5). We used thiopropyl Sepharose 6B beads to purify Skp monomers containing the thiol fragment obtained from the tag‐transfer reaction and the Cys‐containing OmpA bait. The thiol‐containing proteins were then eluted from the resin, separated by SDS‐PAGE and trypsinised in‐gel (*Method 2*). Alternatively, trypsin digestion was performed on‐bead^27^ and unbound peptides removed by washing the resin prior to elution (*Method 3*). Using *Method 2* the number of modified peptides detected increased >2‐fold (from 6 to 14), whilst *Method 3* yielded a further 2.5‐fold increase in the number of modified peptides identified (from 14 to 35) (Figure 4 d, Figure S13), a ca. 6‐fold increase in detection over *Method 1*. No significant sidechain bias was observed for either crosslinker (Figure S14), consistent with the reactivity of the diazirine‐derived carbenes.^9^, 19b Similar crosslinking sites were observed for OmpA(W7C) and OmpA(T144C) using both crosslinkers (Figures S13,S14). Using these enrichment protocols modifications on Skp were identified all around the internal cavity (Figure 4 c), consistent with OmpA tumbling randomly in Skp.^18^ The ability to enrich crosslinked peptides using our tag‐transfer protocol, combined with the promiscuous reactivity of diazirines, exemplifies the power of the technique to monitor even the most dynamic of protein interfaces (Figure 4 c & d).

In summary, we have demonstrated that Cys‐containing variants of a bait protein conjugated with MTS‐diazirine or MTS‐TFMD‐based tag‐transfer crosslinkers can map PPIs in both well‐defined and dynamic interfaces. Since the location of the crosslink in the bait protein is known and only the transferred tag is considered in downstream analysis, the background from bait peptides is removed and the search space for crosslinked products is reduced. These features are particularly important as target protein size increases. The workflow described is simple to implement, only requiring the appropriate crosslinking reagents, LC‐MS/MS and proteomics software for mapping PTMs. Enrichment and digestion of modified peptides enables low protein concentrations to be used, minimising the possibility of aggregation or other aberrant interactions. Additionally, the custom UV LED platform enables PI‐XL on a 10 second timescale, not possible with arc‐based lamps, and only previously achieved using pulsed lasers in solution or in the gas phase.^5^, 10a The low cost (≈$300) and simplicity of our UV LED system makes these timescales accessible to any researcher. Our workflow thus opens the door to time‐resolved XL on the second timescale without the need for expensive lasers and enables the study of conformational changes within dynamic protein complexes versus time.

## Conflict of interest

The authors declare no conflict of interest.

## Supporting information

As a service to our authors and readers, this journal provides supporting information supplied by the authors. Such materials are peer reviewed and may be re‐organized for online delivery, but are not copy‐edited or typeset. Technical support issues arising from supporting information (other than missing files) should be addressed to the authors.

Supplementary
