# Supplementary material for: Rapid Mapping of Protein Interactions Using Tag‐Transfer Photocrosslinkers
Source: Angew Chem Int Ed Engl. 2018 Nov 21;57(51):16688–92. doi: 10.1002/anie.201809149 (PMC6348423; doi:10.1002/anie.201809149)
Supplement: Supplementary file 1 — Supplementary [file ANIE-57-16688-s001.pdf]

Angewandte Chemie
Eine Zeitschrift der Gesellschaft Deutscher Chemiker

Supporting Information

Rapid Mapping of Protein Interactions Using Tag-Transfer Photocrosslinkers

Jim E. Horne+, Martin Walko+, Antonio N. Calabrese+, Mark A. Levenstein, David J. Brockwell, Nikil Kapur, Andrew J. Wilson, and Sheena E. Radford**

anie_201809149_sm_miscellaneous_information.pdf

Author Contributions

J.H. Conceptualization: Equal; Formal analysis: Equal; Investigation: Equal; Methodology: Equal; Writing—original draft: Equal; Writing—review & editing: Equal
M.W. Conceptualization: Equal; Formal analysis: Equal; Investigation: Equal; Methodology: Equal; Writing—original draft: Equal; Writing—review & editing: Equal
A.C. Conceptualization: Equal; Formal analysis: Equal; Investigation: Equal; Methodology: Equal; Writing—original draft: Equal; Writing—review & editing: Equal
M.L. Conceptualization: Equal; Investigation: Equal; Methodology: Equal; Writing—original draft: Equal; Writing—review & editing: Equal
D.B. Conceptualization: Equal; Funding acquisition: Equal; Supervision: Equal; Writing—original draft: Equal; Writing—review & editing: Equal
N.K. Conceptualization: Equal; Funding acquisition: Equal; Methodology: Equal; Supervision: Equal; Writing—original draft: Equal; Writing—review & editing: Equal
A.W. Conceptualization: Equal; Funding acquisition: Equal; Investigation: Equal; Supervision: Equal; Writing—original draft: Equal; Writing—review & editing: Equal.
S.E.R. Conceptualization: Equal; Funding acquisition: Equal; Investigation: Equal; Supervision: Equal; Writing—original draft: Equal; Writing—review & editing: Equal.

Table of Contents

S1. Methods 3

S2. Supplementary Figures 18

Figure S1. Synthesis schemes for (**a**) MTS-diazirine or (**b**) MTS-TFMD. 18

Figure S2: Mechanism of activation and bond-insertion by diazirines via 365 nm illumination................19

Figure S3: Masses and structures of crosslinkers, derivatives, and tags used throughout this methodology 20

Figure S4: Construction and validation of the UV LED device for diazirine crosslinking 21

Figure S5: Fabrication of an acrylic chip with a 33 µL chamber for holding samples 22

Figure S6: Sites of modification on MCL-1 from each BID_{80-102} variant...23

Figure S7: Representative mass spectra of peptides derived from MCL-1 containing the modification associated with tag-transfer from the MTS-diazirine-labelled BID_{80-102} peptide 24

Figure S8: Representative mass spectra of peptides derived from MCL-1 containing the modification associated with tag-transfer from the MTS-TFMD-labelled BID_{80-102} peptide...................................25

Figure S9: Quantification of four residues (L235, H224, V265 and H320) modified in MCL-1 by tag transfer from BID_{80-102} peptides containing the MTS-diazirine tag at different sites 26

Figure S10: Quantification of four residues (I237, M250, F270 and V216) modified in MCL-1 by tag transfer from BID_{80-102} peptides containing TFMD-diazirine at different sites...27

Figure S11: Conjugation efficiencies of MTS-diazirine or MTS-TFMD to OmpA(W7C) or OmpA(T144C) 28

Figure S12: Conjugation of MTS-diazirine or MTS-TFMD to OmpA(W7C) and OmpA(T144C) does not affect folding...29

Figure S13: Sites of modification on Skp from different OmpA mutants, crosslinkers and peptide enrichment strategies...30

Figure S14: Reactivity profile of accessible residues of Skp with MTS-diazirine and MTS-TFMD-conjugated OmpA[Cys] 31

Figure S15: Representative mass spectra of peptides derived from Skp containing the modification associated with tag-transfer from the MTS-diazirine-labelled Cys-OmpA 32

Figure S16: Representative mass spectra of peptides derived from Skp containing the modification associated with tag-transfer from the MTS-TFMD-labelled Cys-OmpA...33

S3. Supplementary Tables 34

Table S1: Affinity (EC50) of labelled BID80-102 peptides to MCL-1 as determined by competition fluorescence anisotropy assay...34

Table S2: MCL-1 Peptides identified with modifications from MTS-diazirine-conjugated BID_{80-102} peptides 35

Table S3: MCL-1 Peptides identified with modifications from TFMD-conjugated BID_{80-102} peptides........36

Table S4: Skp peptides identified with modifications from MTS-diazirine-conjugated T144C and W7C OmpA 37

Table S5: Skp peptides identified with modifications from MTS-TFMD-conjugated T144C and W7C OmpA 40

S4. Supporting References 42

S5. Analytical data for peptides...43

S1. Methods

Organic synthesis

All solvents were purchased from Fisher scientific and all reagents were purchased from Sigma-Aldrich or Fluorochem unless otherwise stated and used without further purification. Purification by column chromatography was carried out using silica gel. Analytical thin layer chromatography (TLC) was conducted using Merck 0.25 mm silica gel pre-coated aluminium plates with fluorescent indicator active at UV245. ^{1}H and ^{13}C and ^{19}F NMR spectra were acquired on Bruker Avance III HD 400 series spectrometer at 400 MHz for ^{1}H, 100 MHz for ^{13}C and 376 MHz for ^{19}F. Chemical shifts are expressed as parts per million using solvent as internal standard ($CDCl_3$ 7.26 ppm in ^{1}H and 77.16 ppm in ^{13}C spectra) and coupling constants are expressed in Hz. The following abbreviations are used: s for singlet, d for doublet, t for triplet, q for quartet, p for pentet, m for multiplet and br for broad.

2-(3-Methyl-3H-diaziren-3-yl)ethanol (1)

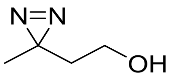

NH_3 (approx.. 100 mL) was condensed, using a dry ice/acetone condenser, into a flask containing 4-hydroxy-2-butanone (15.2 mL, 170 mmol) and cooled to -78 °C. After refluxing (-30 °C bath temperature) for 5 h, hydroxylamine-O-sulfonic acid (21.15 g, 187 mmol) dissolved in MeOH (150 mL) was added at -78 °C and the reaction mixture was allowed to heat to room temperature overnight. The resulting mixture was filtered, the solid residue was washed with MeOH (2 x 20 mL), and the filtrate was concentrated to about 100 mL. Triethylamine (26 mL, 187 mmol) was added to the resulting solution followed by iodine in several portions while cooling the reaction mixture in ice. After adding 29.2 g (115 mmol) of I_2, the colour of iodine persisted, indicating the end of the reaction. The solvents were carefully removed from the reaction mixture (25 °C and 120 mbar) and the residue was partitioned between Et_2O (200 mL) and brine (200 mL) containing sat. aq. $Na_2S_2O_3$ (10 mL). The organic layer was separated and the aqueous layer was extracted with Et_2O (2 x 100 mL). The combined organic extracts were dried over Na_2SO_4 and concentrated to give crude diazirine **1**, which was purified by column chromatography (SiO_2, pentane/Et_2O 1/1) to give 8.64 g (50.8 %) of product as a colourless liquid.

δ_H (400 MHz, $CDCl_3$) 1.08 (s, 3H, CH_3), 1.45 (bs, 1H, OH), 1.64 (t, 2H, J = 6.3 Hz, CH_2), 3.54 (t, 2H, J = 6.3 Hz, CH_2)

3-(2-Iodo-ethyl)-3-methyl-3H-diazirine (2)

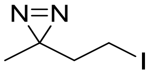

Iodine (3.046 g, 12 mmol) was added to a solution of Ph_3P (3.144 g, 12 mmol) and imidazole (1.634 g, 24 mmol) in dichloromethane (30 mL) at 0 °C. After 15 min stirring, the alcohol **1** (1 g, 10 mmol) was added and the mixture was stirred for 4 h at room temperature. Water (20 mL) was added to the reaction mixture, the organic layer was separated and the aqueous layer was extracted with dichloromethane (2 x 30 mL). The combined organic extracts were dried over Na_2SO_4 and concentrated. The residue was purified by column chromatography (SiO_2, hexane/Et_2O 10/1) to give 1.26 g (60.0 %) of product **2** as a colourless oil.

δ_H (400 MHz, $CDCl_3$) 1.07 (s, 3H, CH_3), 2.02 (t, 2H, J = 7.6 Hz, CH_2), 2.94 (t, 2H, J = 7.6 Hz, CH_2)

S-(2-(3-methyl-3H-diazirin-3-yl)ethyl) methanesulfonothioate (3)

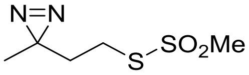

Sodium methanethiosulfonate (160 mg, 1.2 mmol) was added to a solution of iodide **2** (210 mg, 1 mmol) in DMF (1 mL) and the resulting solution was heated at 50 °C for 4 h. After evaporation of the solvent, the residue was purified by column chromatography (SiO_2, hexane/ethyl acetate 3/1) to give 180 mg (92.7 %) of **3** as a colourless oil.

δ_H (400 MHz, $CDCl_3$) 1.09 (s, 3H, CH_3CN_2), 1.85 (t, 2H, J = 7.6 Hz, CH_2CN_2), 3.02 (d, 2H, J = 7.6 Hz, CH_2S), 3.33 (s, 3H, CH_3SO_2); δ_C (100 MHz, $CDCl_3$) 19.81, 24.92, 30.82, 34.95, 50.79;

ESI-HRMS found *m/z* 217.0075 $[M+Na]^+$ $C_5H_{10}N_2NaO_2S_2$ expected 217.0081.

IR: ν_{max}/cm^{-1} (oil) = 2932, 1313, 1130, 956, 744

UV-vis: $\lambda_{max}(\varepsilon)$ (CH_3CN) = 194 (4577), 271 (315), 348 (80)

4-methyl-2,2,2-trifluoroacetophenone (4)

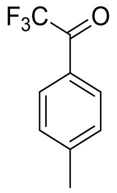

Trifluoroacetic anhydride (27.8 mL, 200 mmol) was slowly added to a solution of N,N-dimethylaminopyridine (24.43 g, 200 mmol) and toluene (23.4 mL, 220 mmol) in dichloromethane (300 mL) at 0 °C. After 10 min of stirring, aluminium chloride (66.67 g, 500 mmol) was added in several portions at 0 °C and the resulting mixture was stirred at room temperature for 16 h. Ice (400 g) was slowly added to the reaction mixture over 1 h. The organic phase was separated and washed with sat. aq. $NaHCO_3$ (120 mL) and brine (120 mL), dried over Na_2SO_4 and concentrated carefully (the bp of the product is reported to be 66 °C at 20 mbar). **4** was isolated (34.43 g, 183 mmol, 91.5 %) as a colourless liquid which contains traces of toluene and was used without further purification; δ_H (400 MHz, $CDCl_3$) 2.46 (s, 3H, CH_3), 7.35 (d, 2H, J = 8.0 Hz, ArCH), 7.98 (d, 2H, J = 7.8 Hz, ArCH); δ_F (376 MHz, $CDCl_3$) -71.32.

4-methyl-2,2,2-trifluoroacetophenone oxime (5)

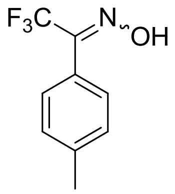

Hydroxylamine hydrochloride (13.90 g, 200 mmol) was added to **4** (34.42 g, 183 mmol) dissolved in the mixture of pyridine (200 mL) and ethanol (100 mL) and the resulting solution was stirred at 60 °C for 4 h. The reaction mixture was concentrated to about 100 mL, diluted with diethyl ether (300 mL) and washed with water (2 x 300 mL) and brine (150 mL). The organic layer was dried over Na_2SO_4 and concentrated to give crude oxime **5** in quantitative yield. This was used in the next step without purification.

4-methyl-2,2,2-trifluoroacetophenone-O-(4-toluenesulfonyl) oxime (6)

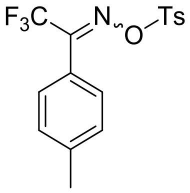

Toluenesulfonyl chloride (45.76 g, 240 mmol) was added to a solution of the crude oxime **5** and trimethylamine (42 mL, 300 mmol) in dichloromethane (300 mL) at 0 °C. The cooling bath was removed and the reaction mixture was stirred for 1 h at room temperature, washed with water (2 x 200 mL) and brine (200 mL), dried over Na_2SO_4 and concentrated to give 66 g of crude toluenesulfonyl oxime **6** as a brown oil that solidifies upon standing. This was used in the next step without purification.

3-(4-methylphenyl)-3-trifluoromethyldiazirine (7)

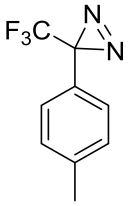

NH_3 (approx. 100 mL) was condensed, using a dry ice/acetone condenser, into the solution of crude tosyl-oxime **6** dissolved in Et_2O (300 mL) and cooled to -78 °C. After refluxing (-30 °C bath temperature) for 5 h, the ammonia was allowed to slowly evaporate overnight. The resulting mixture was filtered, the solid residue was washed with Et_2O (2 x 50 mL), and the filtrate was evaporated. The residue was dissolved in methanol (100 mL) and triethylamine (28 mL, 200 mmol) and iodine was added in several portions while cooling the reaction mixture on ice. After adding 33 g (130 mmol) of I_2, the colour of iodine persisted, indicating the end of the reaction. The solvents were carefully removed from the reaction mixture (25 °C and 120 mbar) and the residue was partitioned between Et_2O (200 mL) and brine (200 mL) containing sat. aq. $Na_2S_2O_3$ (10 mL). The organic layer was separated and the aqueous layer was extracted with Et_2O (2 x 100 mL). The combined organic extracts were dried over Na_2SO_4 and concentrated to give crude diazirine **7**, which was purified by column chromatography (SiO_2, pentane) to give 23.01 g (57.5 %) of product as a colourless liquid.

δ_H (400 MHz, $CDCl_3$) 2.36 (s, 3H, CH_3), 7.09 (d, 2H, *J* = 8.1 Hz, ArCH), 7.20 (d, 2H, *J* = 8.0 Hz, ArCH); δ_C (100 MHz, $CDCl_3$) 21.34, 28.53 (q, *J* = 40.4 Hz), 122.24 (q, *J* = 275 Hz), 126.29, 126.56, 129.67, 139.99 δ_F (376 MHz, $CDCl_3$) -65.42

3-(4-bromomethylphenyl)-3-trifluoromethyldiazirine (8)

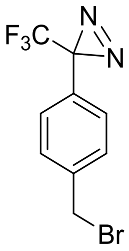

Dibenzoyl peroxide (48 mg, 0.2 mmol), was added to a solution of diazirine **7** (800 mg, 4 mmol) and *N*-bromosuccinimide (854 mg, 4.8 mmol) in dichloroethane (5 mL) and the resulting mixture was stirred at 70 °C for 2 h. The reaction mixture was cooled to room temperature, filtered and the solid washed with dichloromethane (5 mL). The filtrate was evaporated and the residue purified by column chromatography (SiO_2, hexane) to give 990 mg (88.7 %) of **8** as a colourless liquid.; δ_H (400 MHz, $CDCl_3$) 4.46 (s, 2H, CH_2), 7.17 (d, 2H, *J* = 8.1 Hz, ArCH), 7.44 (d, 2H, *J* = 8.4 Hz, ArCH); δ_F (376 MHz, $CDCl_3$) -65.20

***O*-methyl (4-(3-(trifluoromethyl)-3H-diazirin-3-yl)phenyl)methanesulfonothioate (9)**

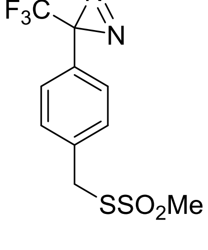

Sodium methanethiosulfonate (160 mg, 1.2 mmol) was added to a solution of bromide **8** (280 mg, 1 mmol) in DMF (1 mL) and the resulting solution was heated at 50 °C for 4 h. After evaporation of the solvent, the residue was purified by column chromatography (SiO_2, hexane/ethyl acetate 3/1) to give 255 mg (82.2 %) of **9** as a colourless oil that solidifies upon standing.

δ_H (400 MHz, $CDCl_3$) 2.98 (s, 3H, CH_3SO_2), 4.37 (s, 2H, CH_2), 7.20 (d, 2H, J = 8.1 Hz, ArCH), 7.44 (d, 2H, J = 8.4 Hz, ArCH); δ_C (100 MHz, $CDCl_3$) 28.38 (q, J = 40.6 Hz), 40.12, 51.34, 121.98 (q, J = 275 Hz), 127.21, 129.35, 129.72, 137.21; δ_F (376 MHz, $CDCl_3$) -65.19

ESI-HRMS found *m/z* 332.9951 $[M+Na]^+$ $C_{10}H_9F_3N_2NaO_2S_2$ expected 332.9955

IR: ν_{max}/cm^{-1} (solid state) = 2928, 1299, 1123, 938, 728

UV-vis: $\lambda_{max}(\varepsilon)$ (CH_3CN) = 200 (22180), 228 (15438), 272 sh (620), 356 (403)

Peptide synthesis

Peptides were synthesised on a CEM Liberty Blue peptide synthesizer with microwave assistance using default coupling cycles. The synthesis was performed on 0.1 mmol scale using Rink Amide MBHA resin (0.33 mmol/g), DMF as a solvent, 20 % (w/v) piperidine in DMF for the deprotection and DIC and OXYMA pure for couplings. Peptides were N-terminally acetylated or fluorescently labelled with fluoresceincarboxylic acid (FAM). Cleavage from resin was accomplished using TFA:H_2O:TIS:EDT, 92.5:2.5:2.5:2.5 (5 mL x 5 h) and peptides were precipitated using cold ether. After preparative HPLC purification using a Jupiter Proteo 90 Å 21.2 x 250 mm reverse phase column and gradient of 20 – 60 % (v/v) acetonitrile with 0.1 % (v/v) TFA and lyophilisation, pure peptides were obtained.

High-resolution mass spectrometry (HRMS) data were recorded using electrospray ionization in positive mode (ESI+) with a Bruker MaXis Impact spectrometer. Analytical HPLC experiments were performed using an Agilent 1290 Infinity LC series system equipped with an Ascentis Express Peptide ES-C18 100 x 2.1 mm column, 2.7 μm particle size on a 5-95 % (v/v) gradient of acetonitrile in water (with 0.1 % (v/v) TFA) over 10 min.

Analytical data for the synthesised peptides, including HRMS and analytical HPLC are shown at the end of the Supplementary Information.

Fluorescence anisotropy assay for BID_{80-102}/MCL-1 binding

The assays were performed in 384 well plates in Tris buffer (50 mM Tris, 150 mM NaCl, 0.01 % (v/v) Triton X-100 pH 7.4) on Perkin Elmer EnVision 2103 MultiLabel plate reader. For the direct titration 25 nM final concentration of FAM labelled BID_{80-102} and MCL-1 with concentration range 50 μM to 0.01 nM were used. For competition titrations, 25 nM final concentration of FAM labelled BID_{80-102}, 150 nM final concentration of MCL-1 and MTS-diazirine labelled BID_{80-102} with concentration range 50 μM to 1 nM were used. The data were processed as outlined in reference[1].

Protein production and purification

Expression and purification of MCL-1

MCL-1 was expressed and purified according to reference[1].

Expression and purification of OmpA and Skp

Cysteine point mutations were introduced into the sequence of full length wild-type mature OmpA using Q5 mutagenesis (New England Biolabs). OmpA, and Skp (containing a hexa-histidine tag) were expressed and purified as described previously[2] with some modifications. After cell disruption, lysate was pelleted (40 mins, 4 °C, 48,000 × *g*) to remove cell debris. The Skp-containing lysate (Skp p*I* ~ 9.7) was applied to 3 x 5 mL HiTrap SP FF cation exchange columns (GE Healthcare) which were washed with start buffer (50 mM $NaPO_4$, 5 mM EDTA, pH 8.0) and Skp eluted over 5 column volumes of elution buffer (1 M NaCl, 50 mM $NaPO_3$, 5 mM EDTA, pH 8.0). Skp-containing fractions as determined by SDS-PAGE were pooled, diluted 1:2, and dialysed overnight (SnakeSkin 3.5K MWCO) into 25 mM Tris-HCl, 150 mM NaCl, 20 mM imidazole, pH 7.6 at 4 °C. The dialysed sample was applied to 3 x 5 mL HisTrap columns (GE Healthcare) which were washed with 25 mM Tris-HCl, 150 mM NaCl, 20 mM imidazole, pH 7.6 and denatured on-column with 25 mM Tris-HCl, 150 mM NaCl, 6 M guanidine-HCl, pH 7.6. Skp was eluted with a 0-500 mM imidazole gradient over 5 column volumes in 25 mM Tris-HCl, 150 mM NaCl, 6 M guanidine-HCl, pH 7.6. Skp-containing fractions were pooled, diluted 1:5 (to 1.2 M guanidine-HCl) and dialysed (SnakeSkin 3.5K MWCO) into 50 mM Tris-HCl, pH 7.2 overnight at 4 °C. The dialysed sample was then applied to 3 x 5 mL Q HP anion exchange columns (GE Healthcare) to bind

impurities. The unbound fraction was retained and concentrated in a 5K MWCO Vivaspin 20 (Sartorius, UK) centrifugal concentrator, aliquoted, snap-frozen in liquid nitrogen and stored at -80 °C.

Labelling with MTS-diazirine or MTS-TFMD

Labelling of $BID_{80\text{-}102}$ Cys-containing peptides

Cysteine-containing peptide (2 µmol) was dissolved in 0.5 mL water and mixed with 0.2 mL DMSO solution of MTS-diazirine label (50 mM). The reaction was completed within 5 min as indicated by LC-MS. The reaction mixture was purified by preparative HPLC under the same conditions as the unlabelled peptides and lyophilised.

Labelling of OmpA Cys mutants

Aliquots of OmpA cysteine mutants were buffer exchanged into 6 M guanidine-HCl, 50 mM Tris-HCl, 10 mM DTT, 1 mM EDTA, pH 8.0 which had been sparged with nitrogen gas and left for 15 min at room temperature to ensure all cysteines are reduced. The presence of EDTA to chelate heavy metal contaminants and the displacement of dissolved O_2 by nitrogen reduce the propensity for the re-oxidation of cysteine or disulfide bond formation before addition of the MTS-based crosslinking reagent. A second buffer exchange was performed into same buffer without DTT. Stock solutions of MTS-diazirine or MTS-TFMD in DMSO were added in 20-times molar excess over OmpA. Final concentrations were: 200-280 µM OmpA, 4 mM MTS-diazirine or MTS-TFMD, 20 % (v/v) DMSO, 4.8 M guanidine-HCl, 40 mM Tris-HCl, and 0.8 mM EDTA. This solution was incubated with rolling at room temperature for 1 hr. The solution was then buffer exchanged using 0.5 mL Zeba™ Spin Desalting Columns, 7K MWCO (Thermo Scientific) into 6 M guanidine-HCl, 50 mM Tris-HCl, 1 mM EDTA, pH 8.0 and stored at 4 °C or aliquoted, snap frozen in liquid nitrogen and stored at -80 °C.

Chip and UV LED Construction

Acrylic chip fabrication

Sample chips for UV irradiation were fabricated from laser-cut poly(methyl methacrylate) (PMMA, Perspex) pieces using a solvent-assisted bonding process developed from Liga *et al.*[3]. Each chip comprises 3 layers: a top UV-transparent layer with inlets/outlets for insertion of a 100 μL pipette tip, a middle layer with a 33 μL well, and a bottom UV-transparent layer which seals the well. Firstly, chip components were designed (**Figure S5c**) and cut from PMMA sheets into 50 x 25 mm^2 pieces with a VLS6.60 CO_2 laser (Universal Laser Systems). Middle pieces were made from 1 mm thick sheets and the top/bottom pieces from 1.8 mm thick sheets. After cutting, these components were rinsed with ethanol and air dried. Next, the pieces were bonded with an Instron 3300 Dual Column Universal Testing System. The system was modified into a heated press using bespoke top and bottom stainless steel plates, each embedded with two cartridge heaters and a thermocouple (**Figure S5a**). Both plates were maintained at 70 °C using a panel mounted proportional-integral-differential (PID) temperature controller (RS Components Ltd). The bottom and middle layers were bonded by pipetting 60 μL of ethanol between them and placing them in the press, which was operated using Bluehill testing software (Instron). Bonding was controlled by a predefined program which compresses the PMMA pieces until a force of 1700 N (1.36 MPa) is reached and held for 2 min. This process is then repeated to bond the top and middle pieces. The stress-strain curve during a typical bonding process can be seen in (**Figure S5b**).

UV LED lamp, optics and sample holders

The heart of the UV lamp was a 4 UV LED array mounted on a standard star circuit board (Led Engin part LZ4-44UV00-0000, peak wavelength 365 nm, radiant flux 4.1 W, viewing angle 110°). This was attached to a heat sink (DragonStar part ILA-HSINK-STAR-50X60MM-BLK-K) to dissipate heat from the LED array. Two additional holes were drilled into the heatsink to allow passage of the electrical wires beneath the mounting plate. A lens was attached to the front of the lamp through the use of a mounting plate (Thorlabs CP02T/M) attached to the heatsink with two threaded bars (M3 thread), to which a lens tube (Thorlabs SM1L20) was attached. A condenser lens (Thorlabs ACL2520U-A, Ø25 mm, f=20.1 mm, NA=0.60), located within this lens tube, was used to focus the light to a 6 mm

spot. As a guide, the centre of the lens was positioned ~20 mm from the LED and ~25 mm from the sample. Power was provided by a dedicated constant current LED controller (eldoLED PWR180D1) regulated to 1000 mA output current. In turn this was powered from a standard desktop power supply with an output of 19 V. The duration of illumination was controlled by a timer-box which controlled a relay that made or broke the circuit between the controller and LED. This approach bypassed the gentle start-up offered by the controller and minimised variation in light intensity. For the chip based experiments, the lamp was mounted conveniently using optical rail (Thorlabs), with a holder used to reproducibly position the chip. For the Eppendorf unit, the lamp was mounted above a block that held the tube.

Measurement of sample heating

Thermocouple measurements of UV LED with microfuge tube

Heating of the solutions due to UV irradiation was investigated using a Pico TC-08 Thermocouple Data Logger. Briefly, a K-type thermocouple was inserted into a think-walled PCR tube containing 200 µL of deionised water. Subsequently, a range of UV exposure times between 5 s and 5 min were conducted during constant temperature monitoring.

Infrared thermography measurements of sample heating

Heating of the wells in the PMMA chips due to the UV LED was monitored by IR Thermography using a FLIR 640 SC camera and ResearchIR software. Images taken directly after exposure times between 5 s and 5 min were utilised to determine the temperature increase, where pixel values corresponding to the well were averaged and compared to their average value before UV exposure. An unheated piece of black electrical tape (emissivity ≈ 1) was used as a reference in each image. The same process was applied to monitor heating of a thin-walled PCR tube containing 200 µL of deionized water after exposure times between 15 s to 20 min from a 6 W 365 nm lamp Hg-Xe lamp.

Cross-linking experiments

Sample preparation

For all irradiation methods, samples were prepared in the following way. For crosslinking of MCL-1/BID_{80-102}, 20 µL of a solution containing MTS-diazirine or MTS-TFMD labelled BID_{80-102} peptide (20 µM) and MCL-1 (20 µM) in buffer 50 mM Tris-HCl, 150 mM NaCl (TBS) pH 7.6 was prepared. Aliquots of Skp trimers were buffered exchanged into TBS, and crosslinker-conjugated OmpA into TBS pH 7.6, 8 M urea using 0.5 mL Zeba Spin Desalting Columns, 7K MWCO (Thermo).

Photo-crosslinking

To form the chaperone-substrate complex, urea-denatured OmpA was diluted into a Skp containing buffer from high (8 M) to low (0.8 M) urea at a final stoichiometry of 2:1 (mol/mol Skp trimer to OmpA monomer (8 µM Skp trimer/4 µM OmpA). For Hg-Xe lamp experiments, 30-130 µL of sample was added to a thin-walled PCR tube and placed on its side on a stack of petri dishes. This was then placed under a covered LF-206.LS 6 W 365 nm lamp (Uvitec) at a distance of 12 mm from the source for different times up to 30 min. For UV LED chip experiments, acrylic chip chambers were washed with TBS pH 7.6 buffer and 33 µL of sample was added. These were then clamped into the chip holder and irradiated with the 365 nm LED for up to 30 s. A pipette was used to remove the sample from the chip via the lower filling hole. For UV LED tube experiments, 30-130 µL of sample was added to a thin-walled PCR tube and placed inside a 0.5 mL microfuge tube which itself was in a 1.5 mL microfuge tube that had been wrapped in aluminium foil (to reflect the UV irradiation within the containers). Samples were irradiated with the 365 nm LED for 30 s. To crosslink MCL-1 BID_{80-102}, samples were irradiated using this apparatus for 1 min.

Separation and enrichment of cross-linked products

To separate and/or enrich crosslinked samples three methods were used as described in the main text.

Method 1

Crosslinked samples were mixed with non-reducing SDS-PAGE loading buffer (final concentrations: 50 mM Tris-HCl pH 6.8, 2 % (w/v) SDS, 0.1 % (w/v) bromophenol blue, 10 % (w/v) glycerol) and run directly on a 15 % (w/v) non-reducing Tris-tricine SDS-PAGE gel to separate cross-linked product from non-cross-linked (or *intra*molecular cross-linked) material. Gels were stained using InstantBlue protein stain (Expedeon). The cross-linked band was then excised for in-gel digestion (see below).

In-gel digestion

Gel bands were cut into approximately 1 mm^3 pieces, and destained by incubating in 30 % (v/v) ethanol at 60 °C for 30 min. Reduction was performed by incubating with 50 µL of 10 mM DTT (in 25 mM ammonium bicarbonate) at 56 °C for 1 hr, followed by alkylation with 50 µL 55 mM IAA (in 25 mM ammonium bicarbonate pH 8) at room temperature for 45 min in the dark. The gel pieces were dehydrated with 100 % acetonitrile, and dried in a laminar flow hood for 60 min. The gel pieces were rehydrated with 20 µL 0.02 $µg.µL^{-1}$ trypsin solution (Promega) in 25 mM ammonium bicarbonate pH 8, and incubated at 37 °C for 18 hr with shaking. Peptides were recovered by incubating gel pieces with 50 µL 60 % (v/v) acetonitrile/5 % (v/v) formic acid (x3) for 10 min. The peptides were then evaporated to dryness and resuspended in 20 µL with 5 % (v/v) acetonitrile/0.1 % (v/v) formic acid prior to MS analysis.

Method 2

30-130 µL of cross-linked sample was buffer exchanged into TBS pH 7.6, 8 M urea, 10 mM DTT using 0.5 mL Zeba™ Spin Desalting Columns, 7K MWCO (Thermo Scientific) and rotated for 30 min at room temperature. This reduced sample was buffer exchanged again into TBS pH 7.6, 8 M urea to remove excess DTT and then added to 200 µL of a thiopropyl Sepharose 6B (GE Healthcare) slurry (100 µL of settled media), mixed by pipetting, and incubated at room temperature for 60 min with rotation. The sample-bound thiopropyl Sepharose beads were pelleted by centrifugation at 14,000 × *g* for 1 min on a bench-top centrifuge and the supernatant removed. The beads were washed with at least double the volume of the media plus sample using TBS pH 7.6, 8 M urea, centrifuged, and the supernatant removed. This step was repeated until no protein was detected in the supernatant by Coomassie stain

after SDS-PAGE analysis (around 6 x 1 mL washes for OmpA-Skp in the concentrations used here). Bound proteins were then eluted by the addition of 100 µL of TBS pH 7.6, 8 M urea, 100 mM β-mercaptoethanol. This was incubated for 30 min at room temperature with rotation before the beads were pelleted by centrifugation and the supernatant retained. The eluted material was separated by SDS-PAGE (see Method 1) and the gels were stained using InstantBlue protein stain (Expedeon). The band corresponding to the target protein (modified with the XL reagent) was then excised for in-gel digestion (see above).

Method 3

Method 3 proceeded in the same way as Method 2 (above), but diverged before the final elution step. Instead of eluting with β-mercaptoethanol, TBS pH 7.6 was added to the beads to dilute the urea concentration to approximately 1 M. Sequencing grade trypsin (Promega) was added to a final 1:50 (w/w) ratio of trypsin:protein and the mixture was incubated at 37 °C, 600 RPM for between 4 hr to overnight. The beads were isolated by centrifugation and the supernatant was discarded. The beads were washed with 2 × 1 mL of TBS pH 7.6, 8 M urea, followed by 5 x 0.5 mL TBS pH 7.6. Bound peptides were eluted by addition of 100 µL of TBS pH 7.6, 8 M urea, 40 mM β-mercaptoethanol and the mixture was incubated with rotation at room temperature for 30 min. The beads were pelleted by centrifugation and the supernatant removed and retained. Free thiols were alkylated by addition of iodoacetamide to a final concentration of 133 mM (50 µL of a 0.4 M stock) and incubated with rotation for 1 hr at room temperature. This sample was then desalted using 10 µL C18 ZipTip pipette tips with 0.6 µL bed volume (Merck) according to manufacturer's instructions. The residual solvent was then evaporated to dryness in a vacuum evaporator (Thermo Scientific). Samples were frozen at -80 °C for later analysis or resuspended in 20 µL 5 % (v/v) acetonitrile/0.1 % (v/v) formic acid for injection onto an LC/MS system.

Mass Spectrometry

Peptides (5 µL) were injected onto a reverse-phase Acquity M-Class C18, 75 µm x 150 mm column (Waters) and separated by gradient elution of 1-50 % solvent B (0.1 % (v/v) formic acid in acetonitrile) in solvent A (0.1 % (v/v) formic acid in water) over 60 min at 300 nL.min^{-1}. The eluate was either infused

either into a Xevo G2-XS (Waters) or Orbitrap Q Exactive (Thermo) mass spectrometer operating in positive ion mode. In all cases, data processing and modification localization was performed using PEAKS Studio 7 (Bioinformatics Solutions). Quantification of modification sites was performed using XCalibur or MassLynx, by generating extracted ion chromatograms for unmodified and modified peptides, integrating the area under the curve, and applying the following equation;

$$\%Modified = \frac{I_{Modified}}{\Sigma(I_{Modified} + I_{Unmodified})} \times 100\ \%$$

Where $I_{Modified}$ and $I_{Unmodified}$ are the intensities of the modified and unmodified peptides, respectively.

For the Xevo G2-XS, mass calibration was performed by infusion of aqueous sodium iodide at a concentration of 2 µg/µl. [Glu1]-Fibrinopeptide B (GluFib) was used as a lock mass calibrant with a 0.5 second lock spray scan taken every 30 seconds during acquisition. The lock mass correction factor was determined by averaging ten scans. Data acquisition was performed in DDA mode with a one second MS scan over an m/z range of 350-2000. The four most intense ions in the MS spectrum were selected for MS/MS by CID, each with a 0.5 second scan over an m/z range of 50-2000. The collision energy applied was dependent upon the charge and mass of the selected ion. Dynamic exclusion of 60 seconds was used.

Orbitrap calibration was performed using Ultramark solution (Thermo). Data acquisition was performed in DDA mode and fragmentation was performed using HCD. Each high-resolution full scan (m/z range 500-2000, R=120,000) was followed by high-resolution product ion scans (R=15,000), with a normalised collision energy of 30 %. The 15 most intense ions in the MS spectrum were selected for MS/MS. Dynamic exclusion of 60 seconds was used.

SDS-PAGE analysis and densitometry

SDS-PAGE gels were imaged on a UV Transilluminator (Syngene) and where appropriate to aid visualisation of low intensity cross-linked bands the contrast was increased globally across the gel in

the GeneSys image acquisition software (Syngene). Densitometry analysis was performed in ImageJ. Densitometry data was fitted in Igor Pro 7 (WaveMetrics) to a single exponential equation of the form:

$$y = A \cdot e^{-kt} + c$$

where *A* is the amplitude, *k* is the rate constant, *t* is time and *c* is the y-intercept .

S2. Supplementary Figures

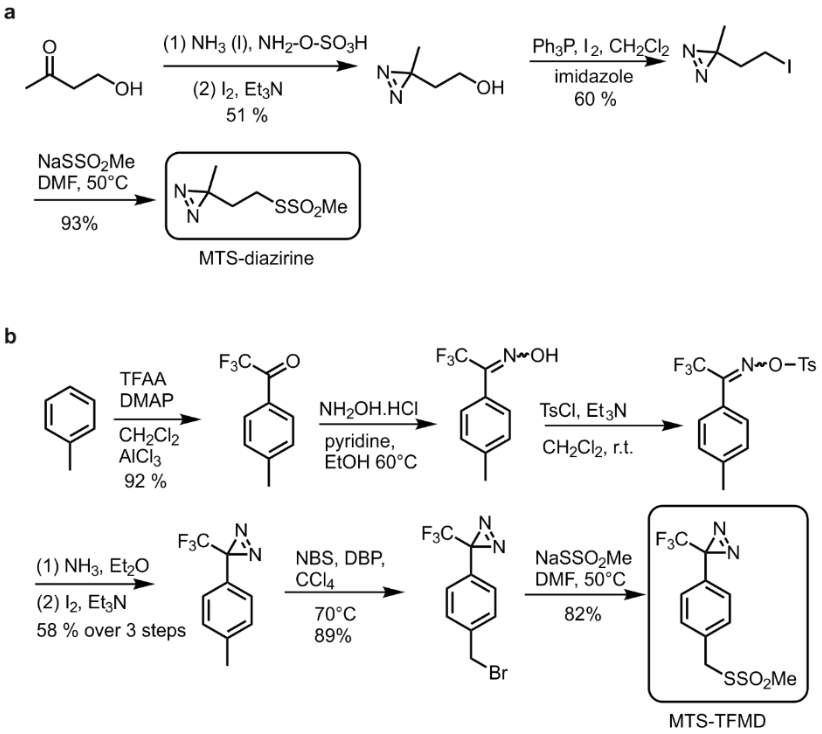

Figure S1. Synthesis schemes for (**a**) MTS-diazirine or (**b**) MTS-TFMD.

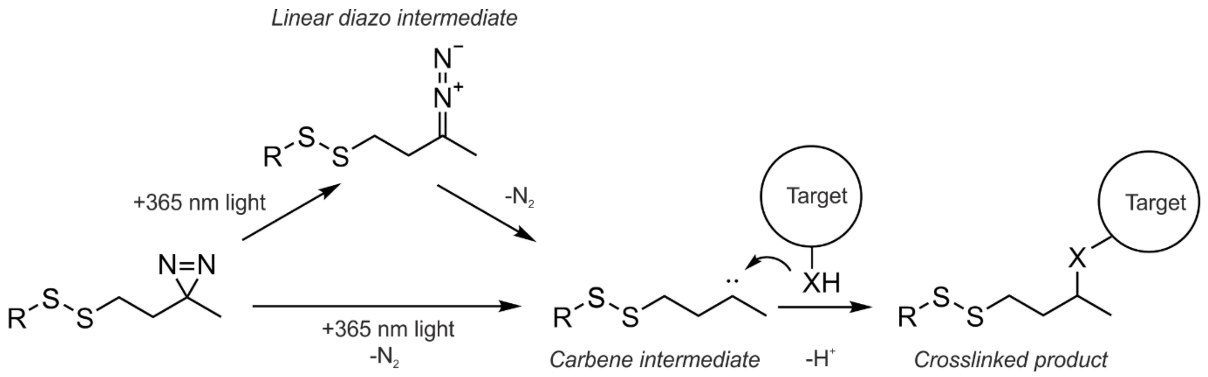

Figure S2: Mechanism of activation and bond-insertion by diazirines via 365 nm illumination. Irradiation of a diazirine moiety with 365 nm light directly unmasks a highly reactive carbene intermediate which will insert non-preferentially into any X-H bond. Alternatively, the reaction can proceed via the formation of a linear diazo intermediate which preferentially reacts with nucleophilic residues.[4]

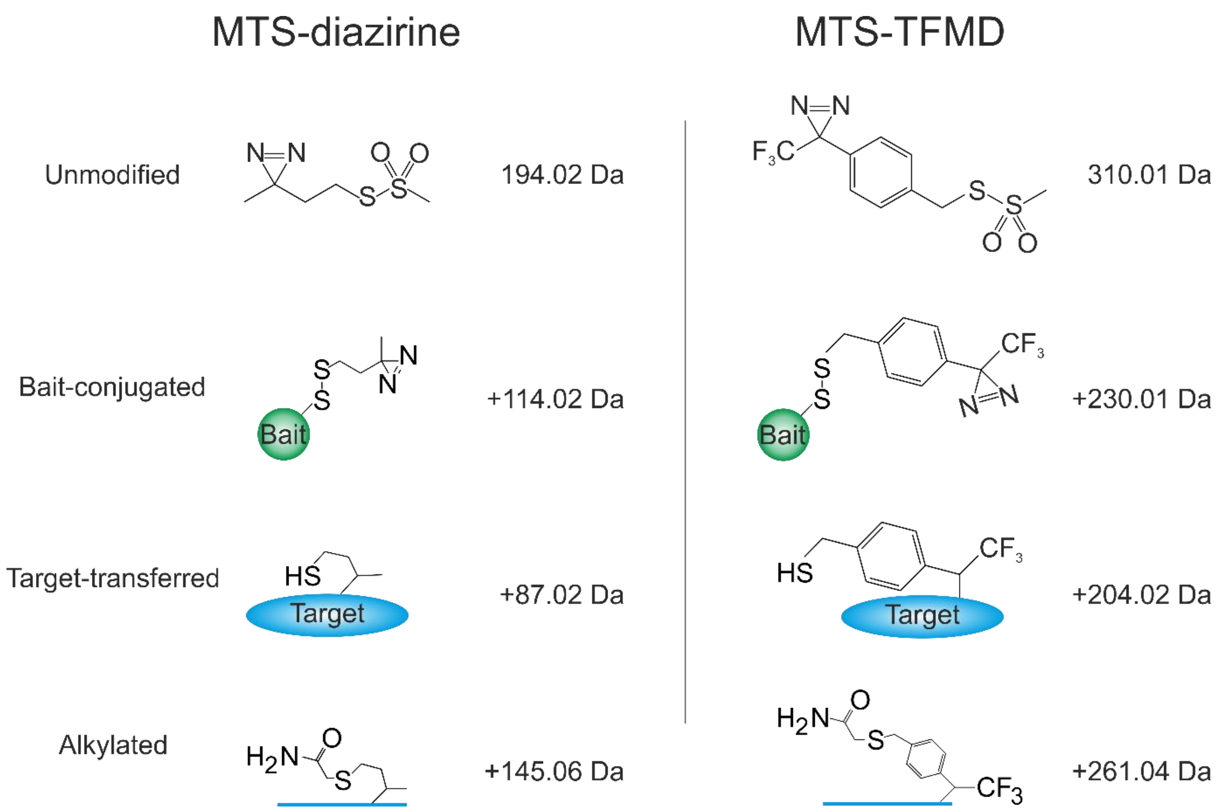

Figure S3: Masses and structures of crosslinkers, derivatives, and tags used throughout this methodology. The raw MTS- and diazirine- containing crosslinker masses and structures are shown as well as the resulting structures and mass adducts after conjugation, tag-transfer, and alkylation steps.

a

Part	Manufacturer	Model Number	Key Parameters
LED	LED ENGIN	LZ4-04UV00	Peak 365nm wavelength, 1 A, 4.10 W
Lens	ThorLabs	ACL2520U-A	Ø25 mm, f=20.1 mm, NA=0.60 ARC: 350-700 nm
LED Driver	eldoLED	PWR180D1	Input voltage: 12-32 V<br>Output current: 1 A
Heat Sink	ILS	ILA-HSINK-STAR-50X60MM-RED-K	–
LED Cage	ThorLabs	CP02T/M	–
Lens Tube	ThorLabs	SM1L10	–

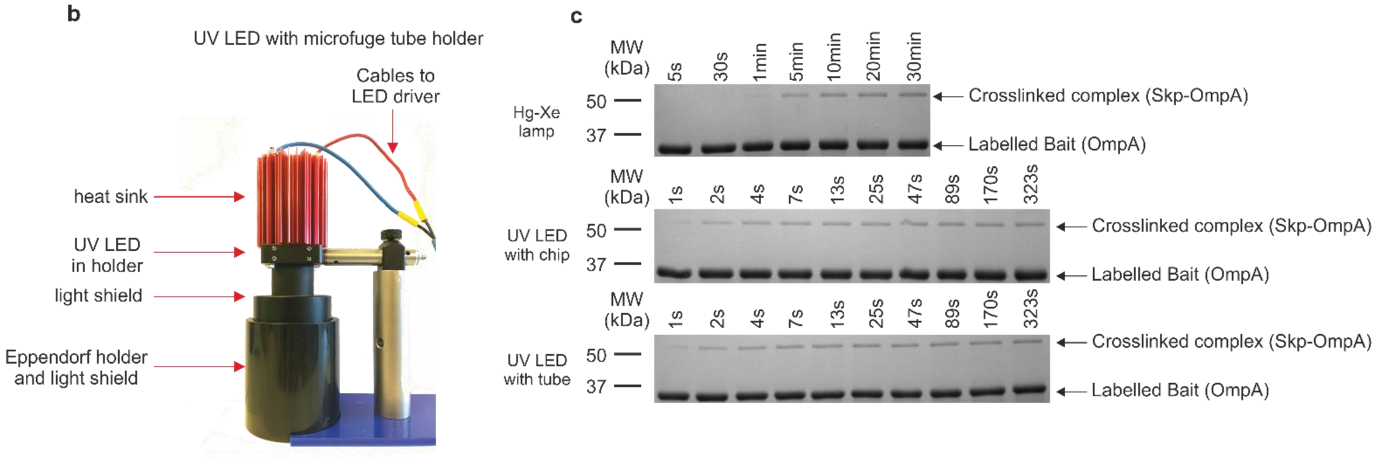

Figure S4: Construction and validation of the UV LED device for diazirine crosslinking. (**a**) List of the components used to construct the UV LED shown in **Figure 1c**. (**b**) The UV LED device for irradiation of samples in microcentrifuge tubes. The UV LED optics are mounted vertically, focussing the UV LED light down the centre of the microcentrifuge tube holder. A 1.5 mL tube (without lid) is placed in the holder and smaller (e.g. 0.5 mL or 0.2 mL) tubes can also be used by nesting them inside each other. The light shield forms a seal around the sample, so additional protective equipment is not required. (**c**) SDS-PAGE analysis of OmpA(W7C)[MTS-diazirine] crosslinking to Skp at increasing irradiation times for a standard Hg-Xe lamp or the UV LED lamp with the sample in a chip or microcentrifuge tube designed in this work. Note the different timescales. Quantification of these data are shown in **Figure 2a**.

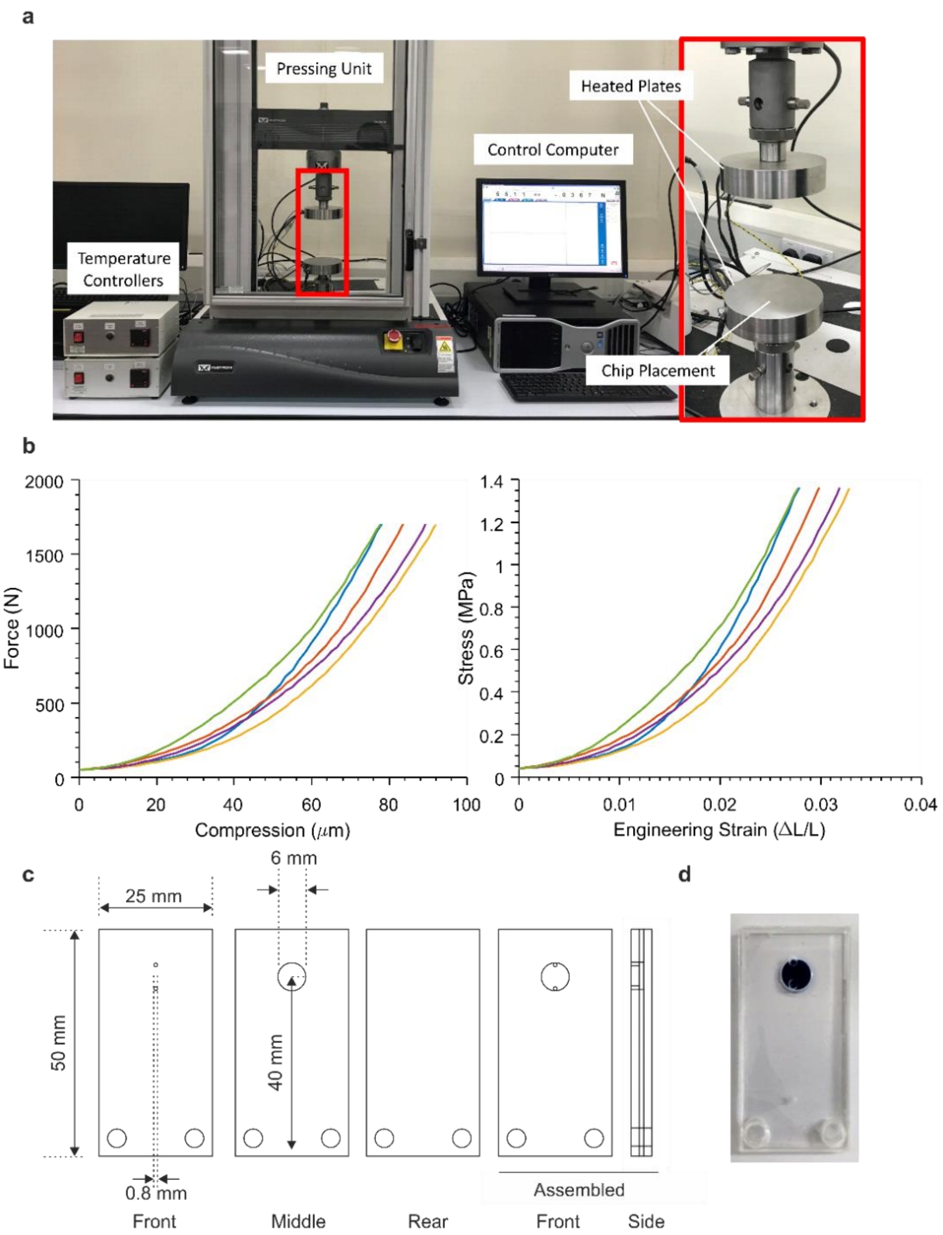

Figure S5: Fabrication of an acrylic chip with a 33 μL chamber for holding samples. (**a**) Heated press setup for bonding laser cut sections of acrylic together into a chip. The press is pre-heated to 70 °C and two chip sections are placed on the lower press with a 60 μL layer of 100 % ethanol in between them. The pieces are then compressed until a force of 1700 N is reached and held for 2 minutes using an Instron 3300 Dual Column Universal Testing System to bond sections together. (**b**) Force-compression (left side) and stress-strain (right side) curves of the initial loading of two acrylic pieces during ethanol-assisted bonding with a heated press. As the press compresses the acrylic sections together, the resulting force is measured by the Instron system. These values can also be converted into the stress and strain experienced by the sections during loading by taking into account the initial thickness and dimensions of the sections. Five individual loading events are shown as different coloured lines to demonstrate reproducibility, where the curves were normalised with a 50 N pre-load. (**c**) Schematic of the three acrylic sections bonded together to form the acrylic chip. A schematic of the assembled chip is shown on the right hand side. (**d**) Photograph of the acrylic chip with the sample well filled with a blue dye solution.

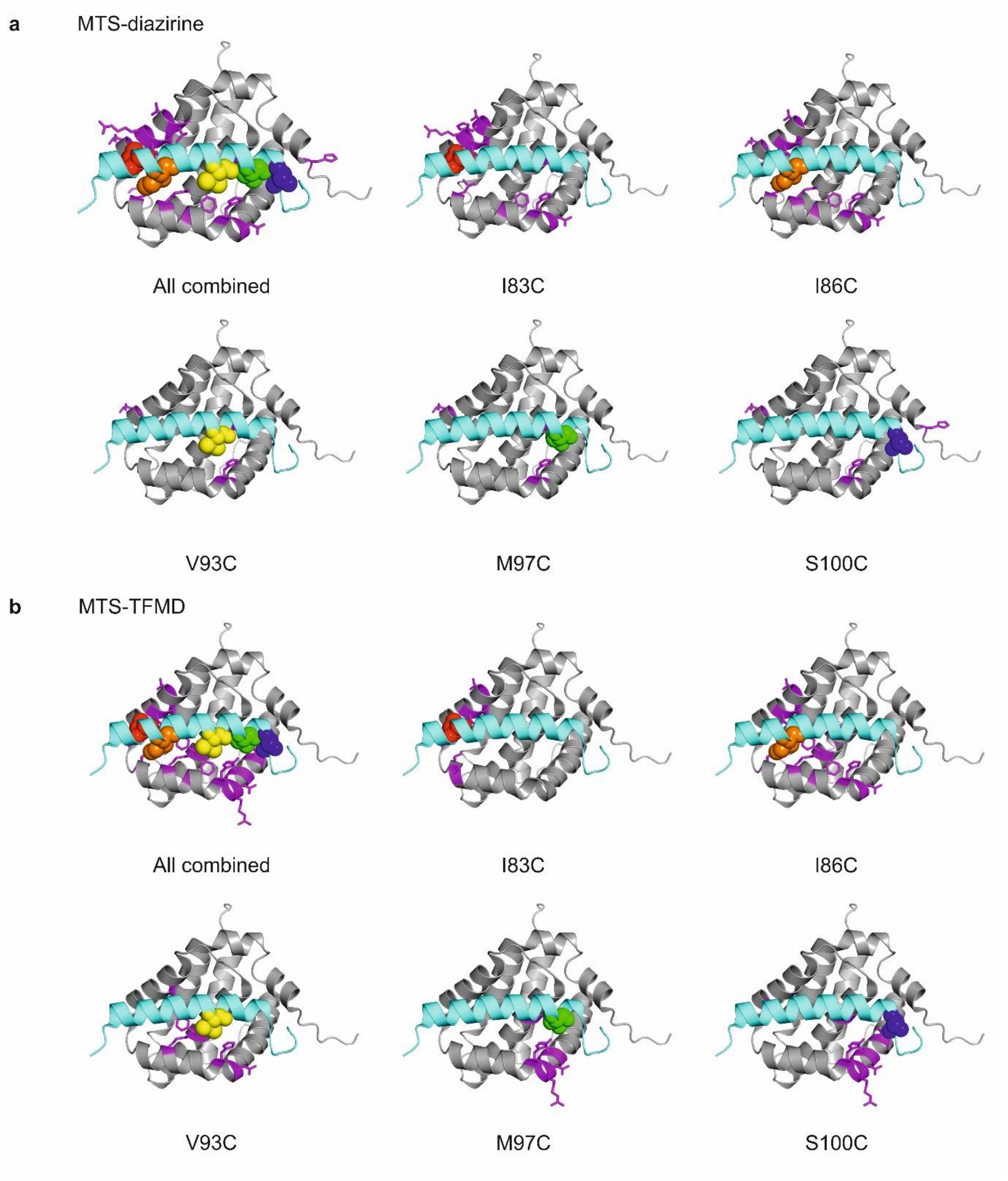

Figure S6: Sites of modification on MCL-1 from each BID_{80-102} variant. Modified sites (magenta), MCL-1 (grey ribbon), BID_{80-102} (cyan), with each residue labelled with each diazirine, as indicated, coloured in space fill. The figures were drawn from the MCL-1:BID NMR structure (PDB ID 2KBW[5]).

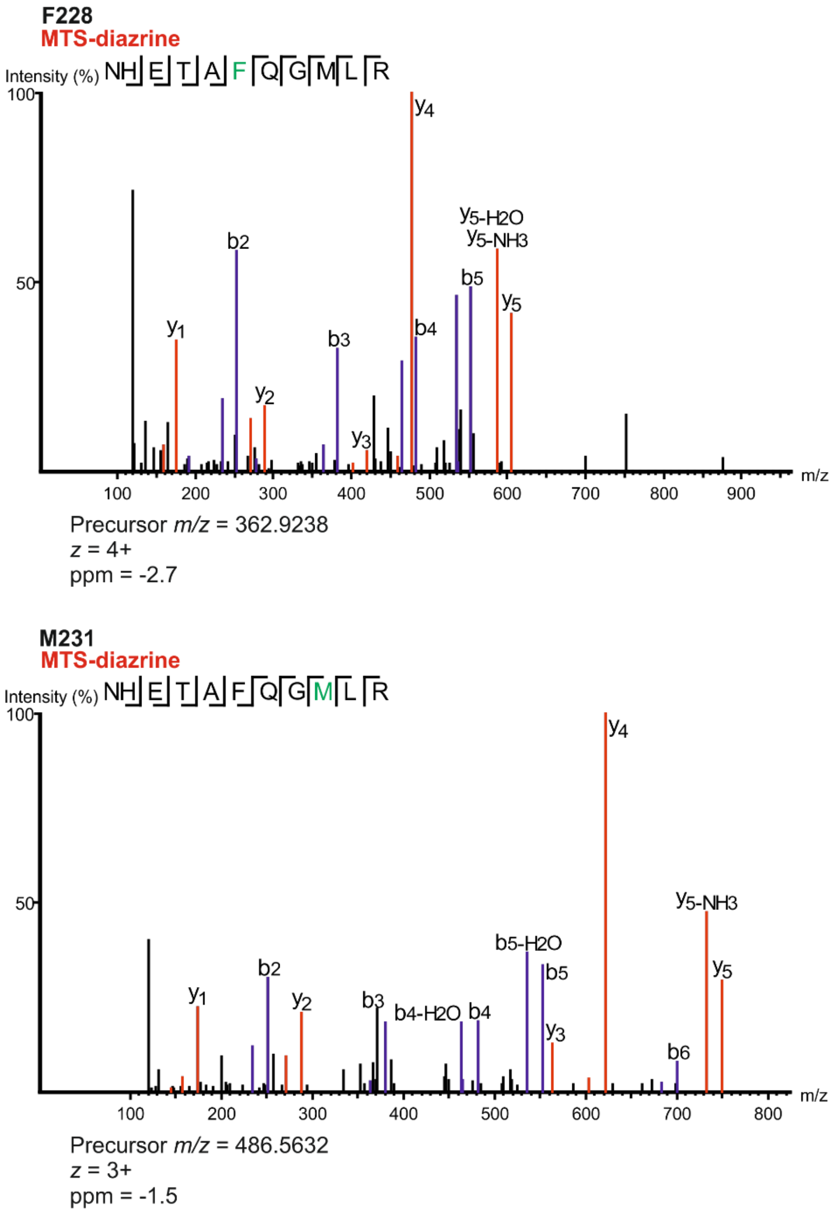

Figure S7: Representative mass spectra of peptides derived from MCL-1 containing the modification associated with tag-transfer from the MTS-diazirine-labelled BID_{80-102} peptide. The site of modification is indicated above the spectrum and in green in the peptide sequence. The precursor *m/z*, charge state and mass error (ppm) are shown.

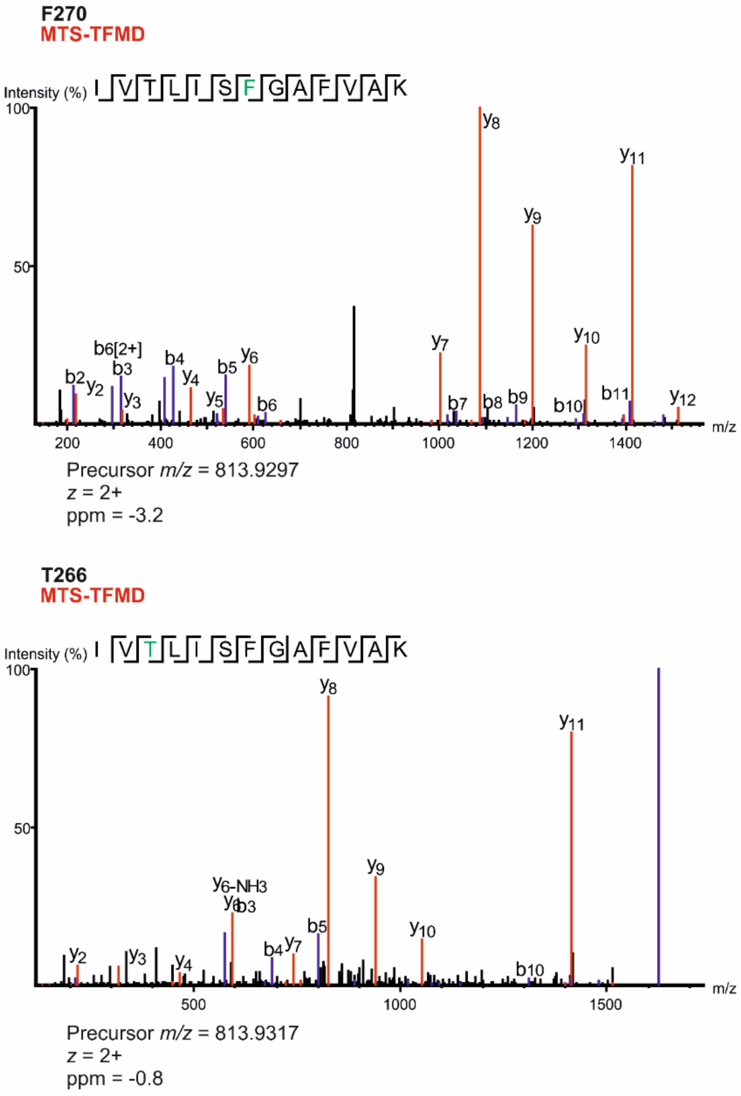

Figure S8: Representative mass spectra of peptides derived from MCL-1 containing the modification associated with tag-transfer from the MTS-TFMD-labelled $BID_{80\text{-}102}$ peptide. The site of modification is indicated above the spectrum and in green in the peptide sequence. The precursor *m/z*, charge state and mass error (ppm) are shown.

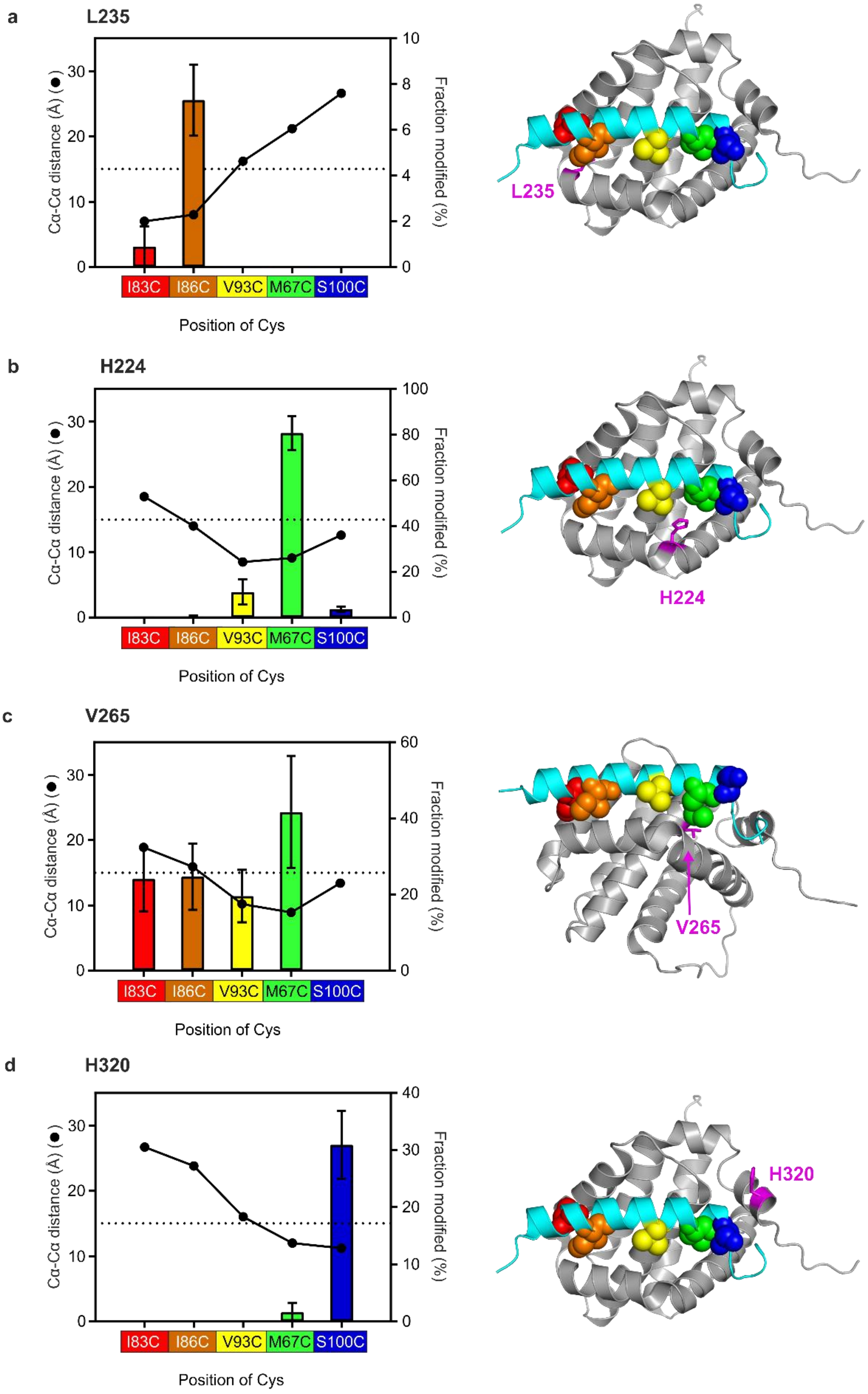

Figure S9: Quantification of four residues (L235, H224, V265 and H320) modified in MCL-1 by tag transfer from BID_{80-102} peptides containing the MTS-diazirine tag at different sites (shown in space fill on the right, with the same colour code used in **a-d**). The percent modification for each residue was quantified (bar charts, right axes) for all five BID_{80-102} peptides. The position of each residue in MCL-1 is shown in magenta in the structure on the right. The Cα-Cα Euclidean distance calculated from the solution structure (PDB ID 2KBW[5]) for each pair is also plotted (data points, left axes), with the dotted line corresponding to a 15 Å distance, as crosslinks were only detected below this distance (apart from V265 which is located directly in the binding groove). Data are shown as mean ± standard error of the mean of two independent experiments.

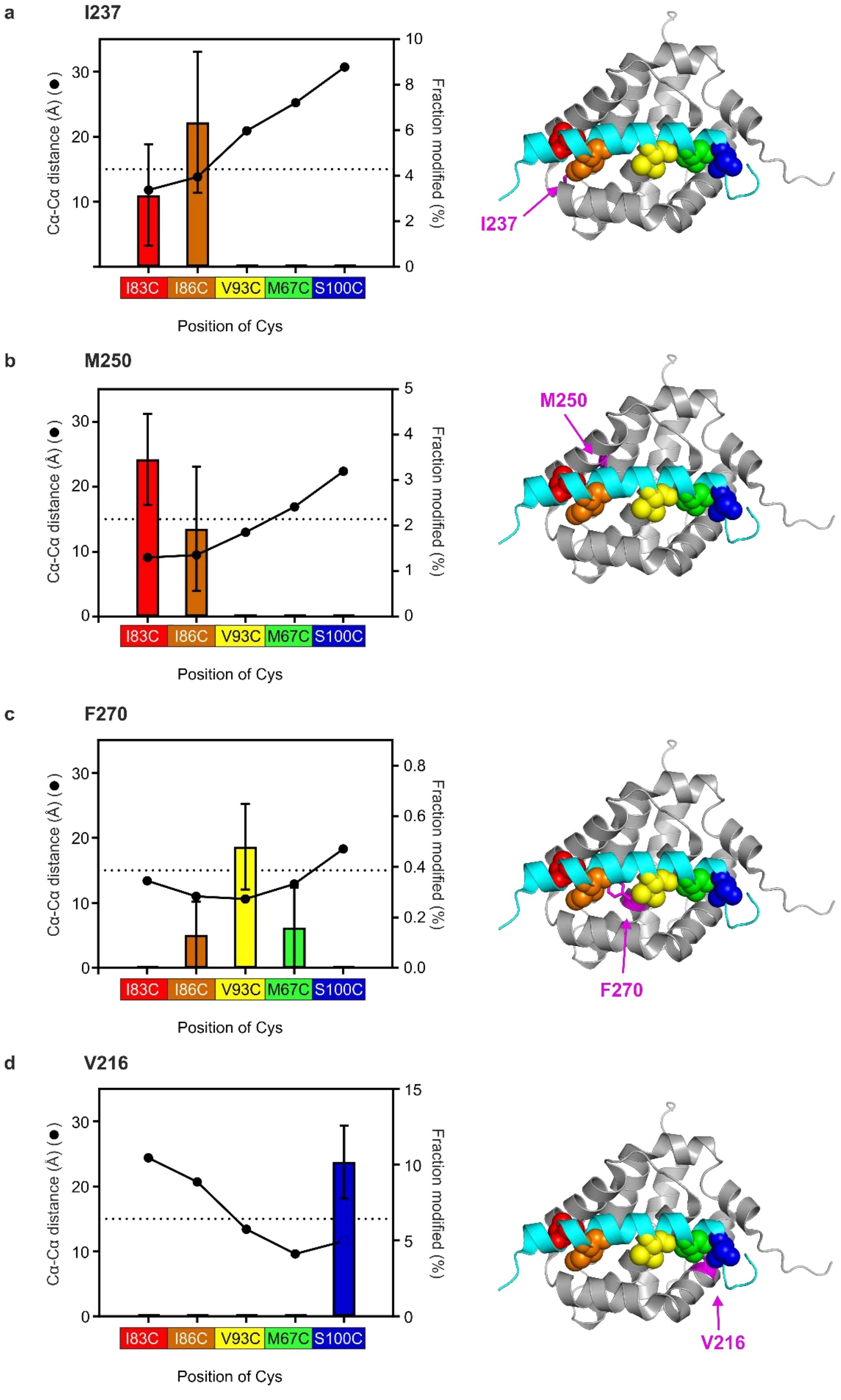

Figure S10: Quantification of four residues (I237, M250, F270 and V216) modified in MCL-1 by tag transfer from $BID_{80\text{-}102}$ peptides containing TFMD-diazirine at different sites (shown in space fill on the right, with the same colour code used in **a-d**). The percent modification for each residue was quantified (bar charts, right axes) for all five $BID_{80\text{-}102}$ peptides. The position of each residue in MCL-1 is shown in magenta in the structure on the right. The Cα-Cα Euclidean distance calculated from the crystal structure for each pair is also plotted (data points, left axes), with the dotted line corresponding to a 15 Å distance, as crosslinks were only detected below this distance. Data are shown as mean ± standard error of the mean of two independent experiments.

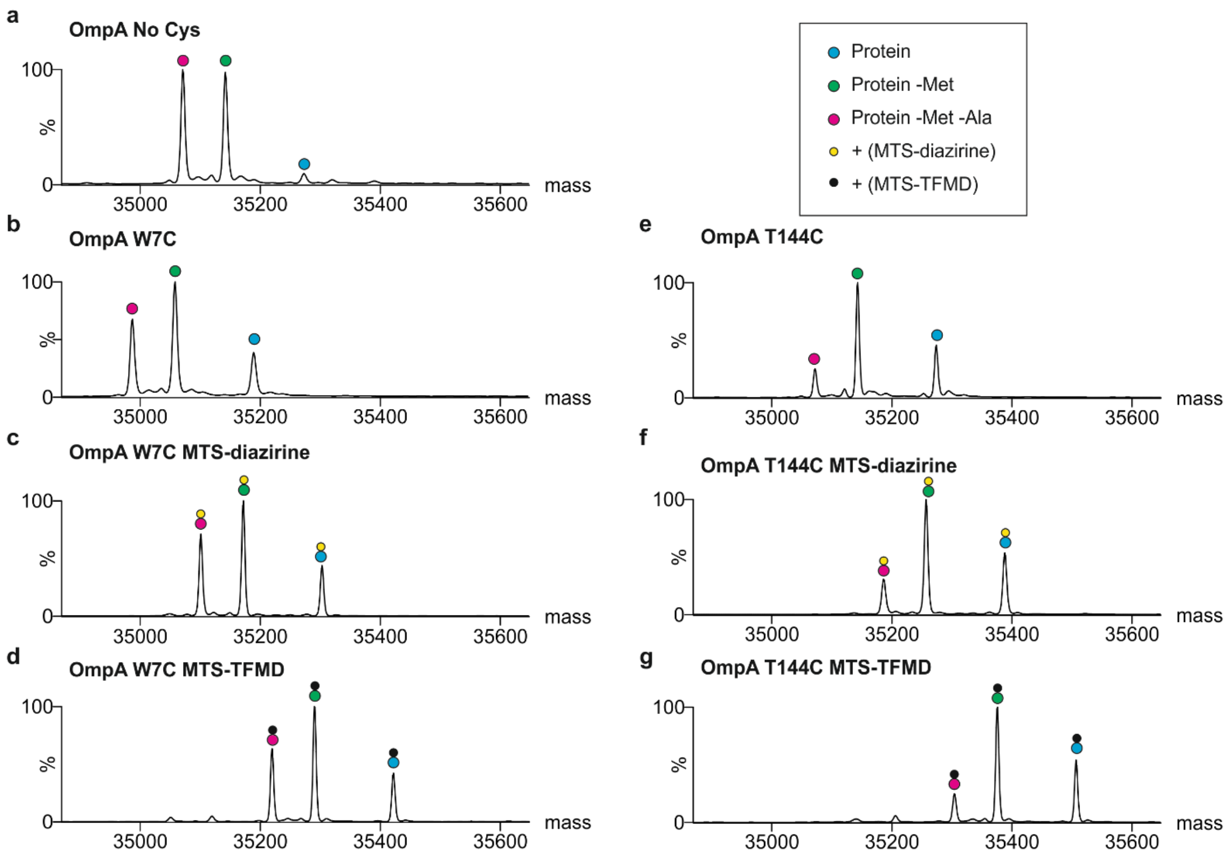

h

	Expected mass (Da)			Observed mass (Da)		
	Protein	- Met	- Met -Ala	Protein	- Met	- Met -Ala
OmpA NoCys	35271	35140	35069	35272	35142	35071
OmpA W7C	35188	35057	34986	35189	35058	34987
OmpA T144C	35273	35142	35071	35274	35143	35072
OmpA W7C + MTS-diazirine	35302	35171	35100	35303	35172	35101
OmpA T144C +MTS-diazirine	35387	35256	35185	35388	35257	35186
OmpA W7C + MTS-TFMD	35418	35287	35216	35422	35290	35220
OmpA T144C +MTS-TFMD	35503	35372	35301	35507	35378	35304

MTS-diazirine = +114 Da
MTS-TFMD = +230 Da

Figure S11: Conjugation efficiencies of MTS-diazirine or MTS-TFMD to OmpA(W7C) or OmpA(T144C). Cysteine mutants of OmpA were labelled with either MTS-diazirine or MTS-TFMD. Labelling reactions were performed for 1 hr at room temperature, and intact MS was performed to determine the presence of labelled protein. (**a**) The deconvoluted mass distribution of OmpA purified from inclusion bodies shows variable loss of the N-terminal two residues (Met and Ala). (**b-d**) Deconvoluted mass distributions of (**b**) OmpA W7C, and OmpA W7C labelled with (**c**) MTS-diazirine or (**d**) MTS-TFMD. (**e-g**) Deconvoluted mass distribution spectra of (**e**) OmpA T144C, and OmpA T144C labelled with (**f**) MTS-diazirine or (**g**) MTS-TFMD. The distribution of peak intensities shows that 100 % labelling of both Cys variants of OmpA was achieved. (**h**) Table of expected and observed masses for the unlabelled and labelled OmpA proteins.

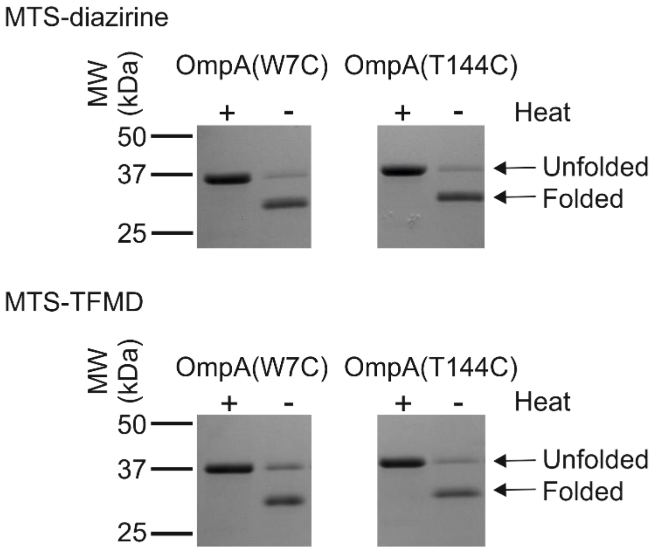

Figure S12: Conjugation of MTS-diazirine or MTS-TFMD to OmpA(W7C) and OmpA(T144C) does not affect folding. SDS-PAGE gel of OmpA mutants following incubation in the presence of di-undecanoyl-phosphatidylcholine (DUPC [*di*$C_{11:0}$]) large unilamellar vesicles (LUVs), with or without boiling prior to loading. In unboiled samples, folded OMPs migrate faster on the SDS-PAGE gel than unfolded OMPs. Upon boiling the OMPs unfold. Both OmpA(W7C) and OmpA(T144C) are able to fold into DUPC (*di*$C_{11:0}$) after being conjugated with either MTS-diazirine or MTS-TFMD. Final conditions were 1 μM OmpA, 3200:1 mol/mol lipid-to-protein ratio for DUPC (*di*$C_{11:0}$), 50 mM glycine pH 9.5, 0.24 M urea.

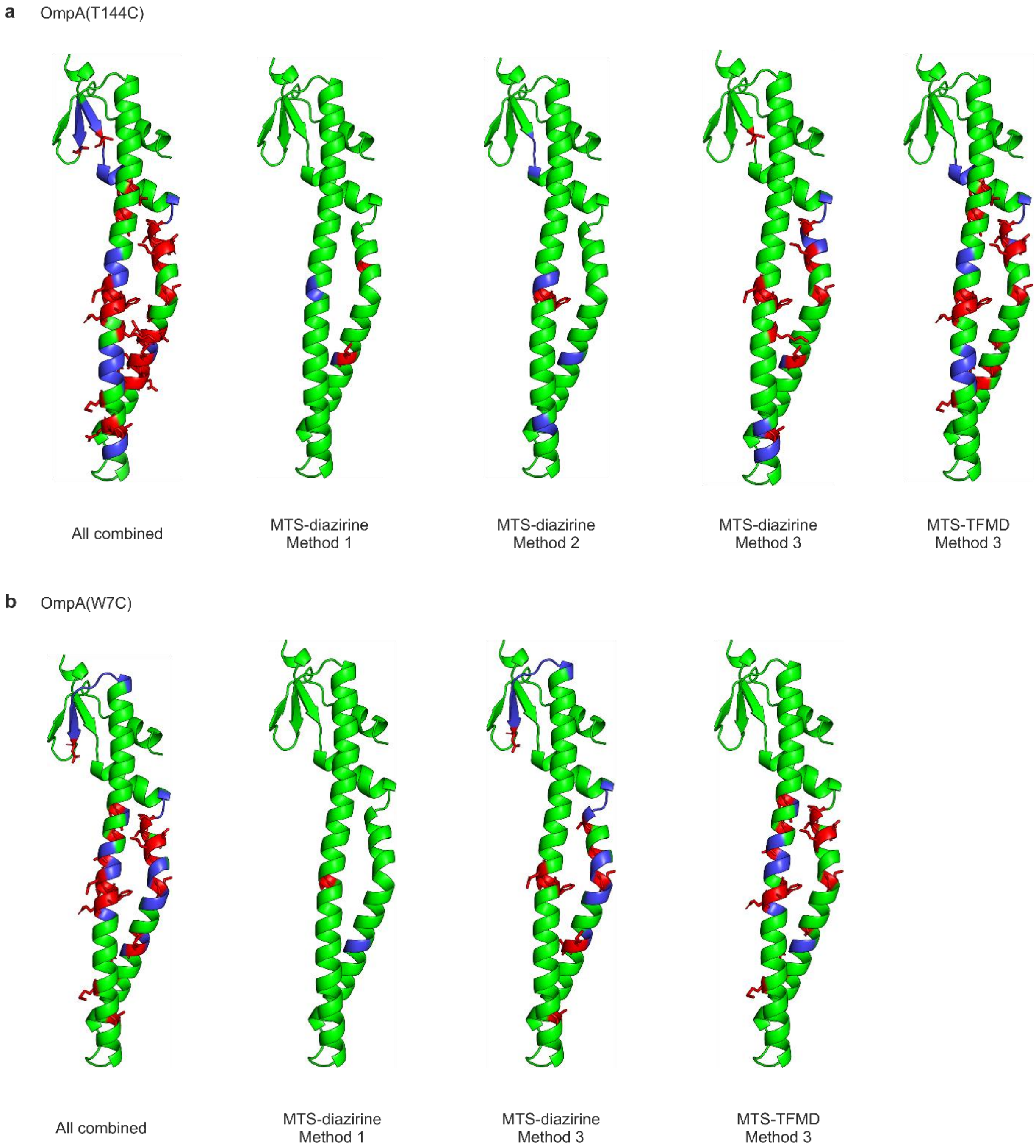

Figure S13: Sites of modification on Skp from different OmpA mutants, crosslinkers and peptide enrichment strategies. Only one monomer is shown in green. For both (**a**) OmpA(T144C) and (**b**) OmpA(W7C), MTS-diazirine and MTS-TFMD derived crosslinks were detected in the Skp 'tentacles'. The exact crosslink varied in each case, possibly reflecting the dynamics of Skp-bound OmpA. This illustrates the utility of using both crosslinkers in the same experiment to further enrich the number of identified interactions in the dataset. Crosslinks which were confidently identified to a residue-level are indicated as red and shown as sticks on the cartoon backbone. Crosslinks which could only be assigned with confidence to a subpeptide level are indicated as a blue cartoon backbone.

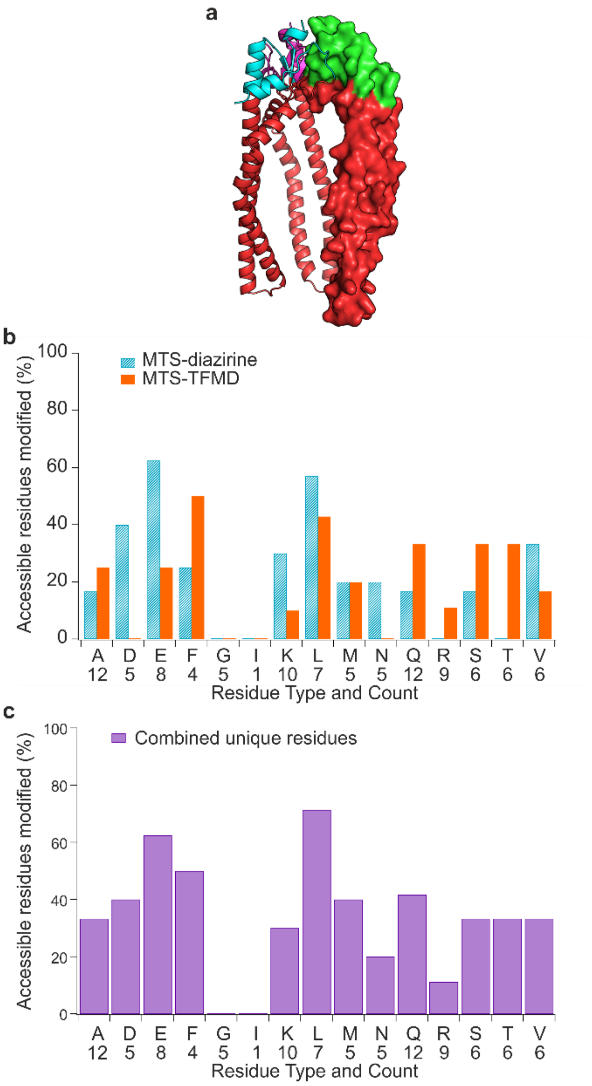

Figure S14: Reactivity profile of accessible residues of Skp with MTS-diazirine and MTS-TFMD-conjugated OmpA[Cys]. (**a**) Based on the literature[6], we made the assumption that the 'crown' region of Skp (green spacefill) is inaccessible to substrate but any part of the cavity or 'tentacles' may come in contact with OmpA, and therefore might be crosslinked. We defined these as 'accessible residues' (comprising the cavity or tentacle region of Skp, corresponding to residues V27-Q121 and D138-A143, as indicated in red, with one 'tentacle' shown in spacefill). (**b**) MTS-diazirine and MTS-TFMD react with different residues in Skp. The percent of each 'accessible residue' type modified was calculated from the number of unique residue-level crosslink assignments. The absolute number of each residue type present in the Skp 'accessible residue' region are shown below each bar. Cysteine, histidine, proline, tyrosine and tryptophan residues are not present in this region of Skp and have been omitted. (**c**) The same data as **b**, but not split by crosslinker type. Note that few Ile and Gly crosslinks were observed, but the former is poorly represented in Skp.

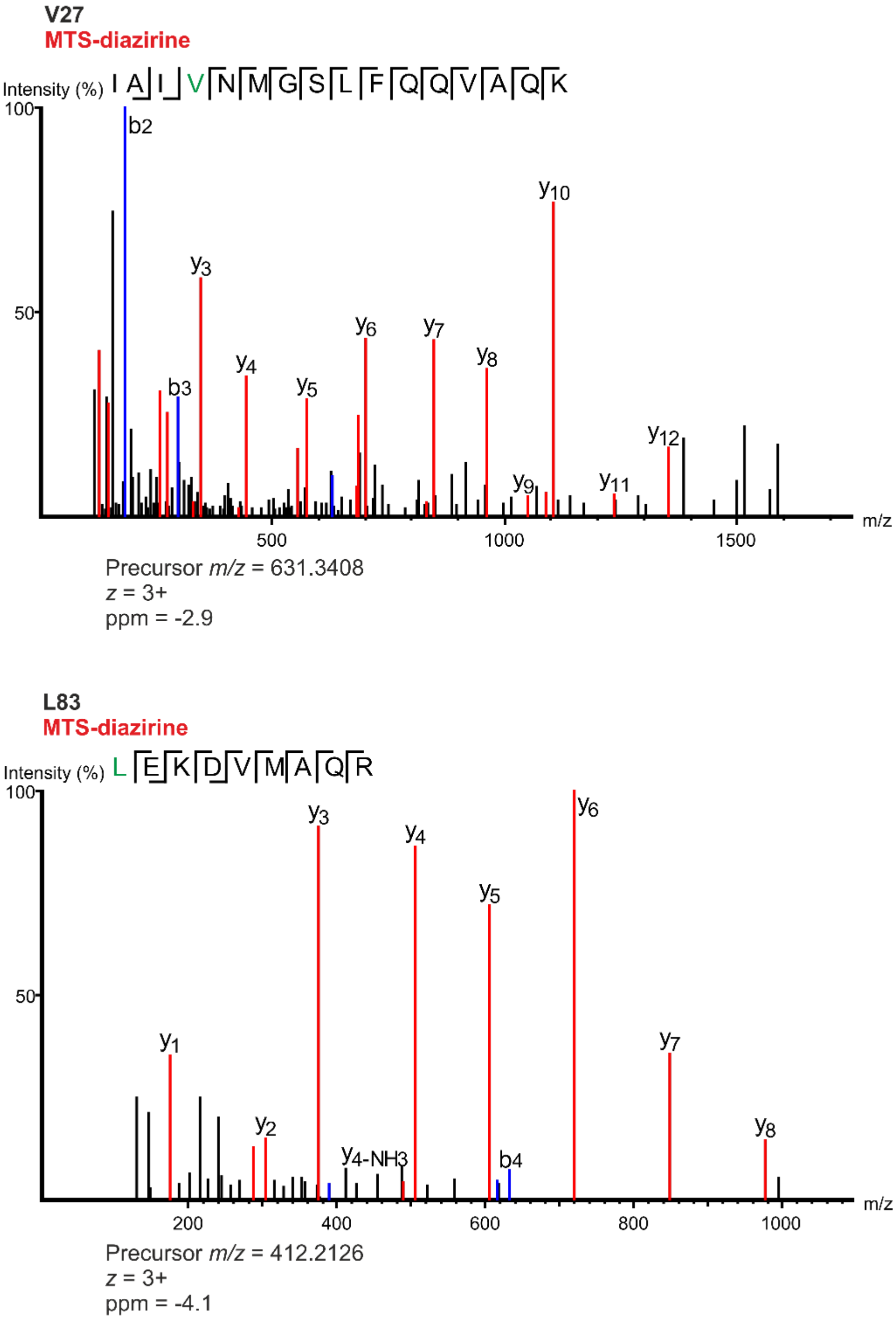

Figure S15: Representative mass spectra of peptides derived from Skp containing the modification associated with tag-transfer from the MTS-diazirine-labelled Cys-OmpA. The site of modification is indicated above the spectrum and in green in the peptide sequence. The precursor *m/z*, charge state and mass error (ppm) are shown.

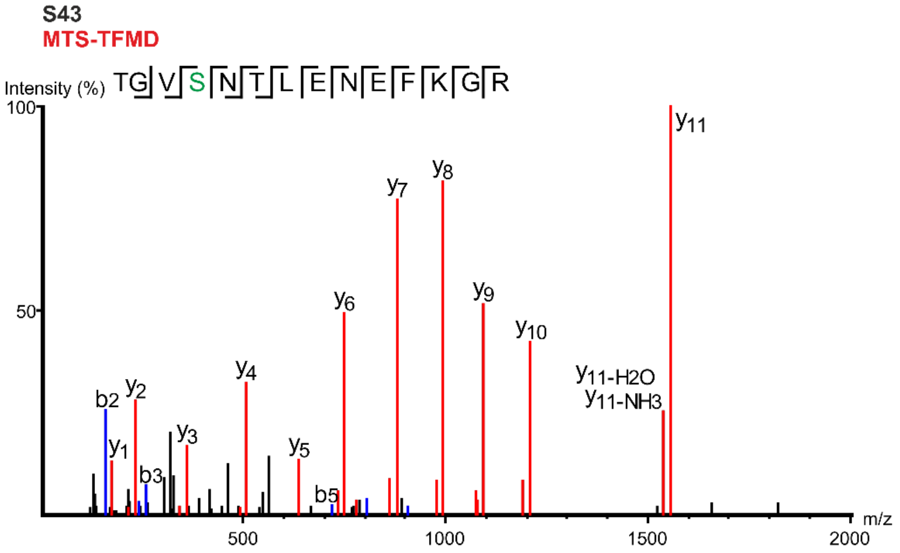

Precursor *m/z* = 906.9106
z = 2+
ppm = -3.0

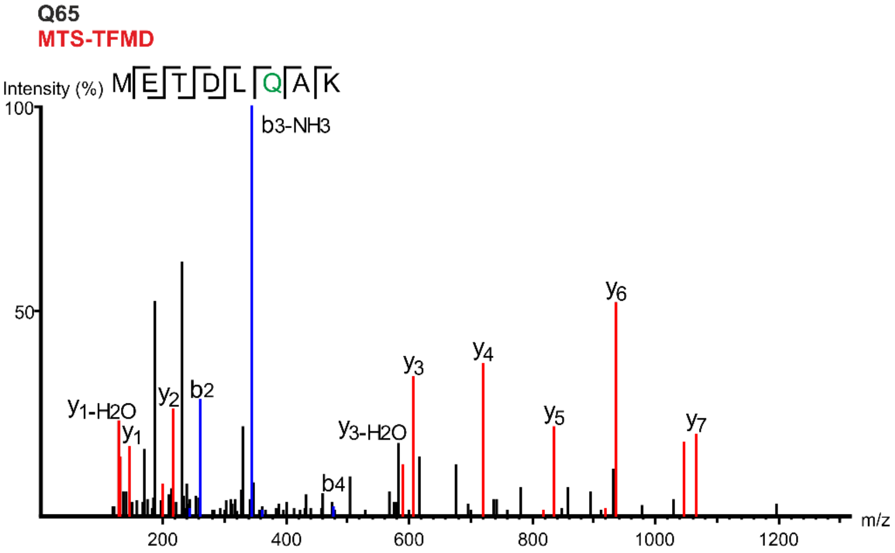

Precursor *m/z* = 598.7498
z = 2+
ppm = -1.0

Figure S16: Representative mass spectra of peptides derived from Skp containing the modification associated with tag-transfer from the MTS-TFMD-labelled Cys-OmpA. The site of modification is indicated above the spectrum and in green in the peptide sequence. The precursor *m/z*, charge state and mass error (ppm) are shown.

S3. Supplementary Tables

Table S1: Affinity (EC50) of labelled BID80-102 peptides to MCL-1 as determined by competition fluorescence anisotropy assay. Crosslinking efficiency of labelled BID_{80-102} peptides to MCL-1 based on the SDS-PAGE band intensity of crosslinked and non-crosslinked MCL-1 shown in **Figure 3c**. The values and standard deviations are results of three repeats. n.d = not determined as binding was too weak to enable an accurate fit to be obtained.

BID peptide	EC_{50} for affinity to MCL-1 (nM)	Crosslinked Mcl-1 (%)
WT	460 ± 19	0
**MTS-diazirine labelled**		
I83C	406 ± 16	19 ± 2
I86C	5506 ± 1128	16 ± 2
V93C	8746 ± 2341	9 ± 3
M97C	422 ± 15	41 ± 6
S100C	360 ± 12	32 ± 4
**MTS-TMFD labelled**		
I83C	338 ± 9	51 ± 6
I86C	1407 ± 53	53 ± 1
V93C	n.d.	40 ± 8
M97C	255 ± 11	26 ± 1
S100C	287 ± 24	31 ± 3

Table S2: MCL-1 Peptides identified with modifications from MTS-diazirine-conjugated BID_{80-102} peptides. Residues found to be modified in MCL-1 are underlined and shown in green. Where the spectral quality of the MS/MS spectra was insufficient to conclusively assign the modified residue, the sub-peptide where the modification is present is indicated.

Sequence	Start Residue	End Residue	Modified Residue	Number of spectra
**I83C**				
**NH**ETAFQHMLR	223	233	223-224	9
NHETA**F**QHMLR	223	233	228	2
K**L**DIKNEDDVK	234	244	235	3
SL**SR**VMIHVFSDGVTNWGR	245	263	247-248	9
**VMI**HVFSDGVTNWGR	249	263	249-251	1
VMI**H**VFSDGVTNWGR	249	263	252	1
**V**TLISFGAFVAK	265	276	265	7
TINQE**S**CIEPLAESITDVLVR	280	300	285	9
**I86C**				
**NH**ETAFQHMLR	223	233	223-224	21
NHETA**F**QHMLR	223	233	228	15
NHETAFQH**M**LR	223	233	231	17
K**L**DIKNEDDVK	234	244	235	17
NEDDV**K**SLSR	239	248	244	1
**VMI**HVFSDGVTNWGR	249	263	249-251	3
TINQE**S**CIEPLAESITDVLVR	280	300	285	17
**V93C**				
N**H**ETAFQHMLR	223	233	224	32
NEDDV**K**SLSR	239	248	244	11
**V**TLISFGAFVAK	265	276	265	1
TINQE**S**CIEPLAESITDVLVR	280	300	285	19
**M97C**				
N**H**ETAFQHMLR	223	233	224	101
NEDDV**K**SLSR	239	248	244	8
**V**TLISFGAFVAK	265	276	265	3
TINQE**S**CIEPLAESITDVLVR	280	300	285	40
**S100C**				
N**H**ETAFQHMLR	223	233	224	31
NEDDV**K**SLSR	239	248	244	2
TINQE**S**CIEPLAESITDVLVR	280	300	285	40
GWDGFVEFF**H**VEDLEGG	311	327	320	21

Table S3: MCL-1 Peptides identified with modifications from TFMD-conjugated BID$_{80-102}$ peptides. Residues found to be modified in MCL-1 are underlined and shown in green. Where the spectral quality of the MS/MS spectra was insufficient to conclusively assign the modified residue, the sub-peptide where the modification is present is indicated.

Sequence	Start Residue	End Residue	Modified Residue	Number of spectra
**I83C**				
**K**LDIKNEDDVK	234	244	234	4
KLD**I**KNEDDVK	234	244	237	3
NED**D**VKSLSR	239	248	242	3
V**M**IHVFSDGVTNWGR	249	263	250	4
TINQE**S**CIEPLAESITDVLVR	280	300	285	4
**I86C**				
**NH**ETAFQGMLR	223	233	223-224	2
NHETA**F**QGMLR	223	233	228	4
NHETAFQG**M**LR	223	233	231	5
KLD**I**KNEDDVK	234	244	237	4
**VMI**HVFSDGVTNWGR	249	263	249-251	15
IVTL**I**SFGAFVAK	264	276	268	5
IVTLIS**F**GAFVAK	264	276	270	5
**V93C**				
**NH**ETAFQGMLR	223	233	223-224	2
NHETAFQG**M**LR	223	233	231	12
V**M**IHVFSDGVTNWGR	249	263	250	3
IV**T**LISFGAFVAK	264	276	266	1
IVTLIS**F**GAFVAK	264	276	270	2
**M97C**				
VGD**GVQR**	216	222	219-222	1
**NH**ETAFQGMLR	223	233	223-224	7
IV**T**LISFGAFVAK	264	276	266	2
**S100C**				
**V**GDGVQR	216	222	216	2
VGD**GVQR**	216	222	219	1
**N**HETAFQGMLR	223	233	223	5
N**H**ETAFQGMLR	223	233	224	2
NEDD**V**K	239	244	243	1
IV**T**LISFGAFVAK	264	276	266	2

Table S4: Skp peptides identified with modifications from MTS-diazirine-conjugated T144C and W7C OmpA. * = oxidation, † = carbamylation, ‡ = deamidation.

Sequence	Start Residue	End Residue	Modified Residue	Number of Spectra	Enrichment	Lamp
**T144C**						
AIVNMGSLFQQVAQK	25	39	25-31	1	Method 1	Hg-Xe
TGVSNTLENEFK	40	51	47	1	Method 1	Hg-Xe
METDLQAK	60	67	60	1	Method 1	Hg-Xe
M*ETDLQAK	60	67	60-61	1	Method 1	Hg-Xe
METDLQAK	60	67	61	1	Method 1	Hg-Xe
AQAFEQDR	98	105	102-103	1	Method 1	Hg-Xe
AQAFEQDR	98	105	102	1	Method 1	Hg-Xe
IAIVNMGSLFQQVAQK	24	39	27	3	Method 2	Hg-Xe
AIVNMGSLFQQVAQK	25	39	27-29	10	Method 2	Hg-Xe
AIVNMGSLFQ‡QVAQK	25	39	27-30	1	Method 2	Hg-Xe
AIVNMGSLFQQVAQK	25	39	27-29	1	Method 2	LED
ASELQ‡R	54	59	57	1	Method 2	Hg-Xe
M*ETDLQAK	60	67	60-61	1	Method 2	LED
M*ETDLQAK	60	67	60	25	Method 2	Hg-Xe
METDLQAK	60	67	60	8	Method 2	Hg-Xe
METDLQAK	60	67	60-61	1	Method 2	Hg-Xe
M*ETDLQAK	60	67	60-61	3	Method 2	Hg-Xe
METDLQ‡AK	60	67	60-61	1	Method 2	Hg-Xe
M*ETDLQ‡AK	60	67	60-62	1	Method 2	Hg-Xe
E†TDLQAK	61	67	61	3	Method 2	Hg-Xe
TK†LEK†DVM*AQR	81	91	84-85	1	Method 2	LED
L†EKDVM*AQR	83	91	83-84	1	Method 2	LED
QTFAQK†AQAFEQDR	92	105	102	2	Method 2	LED
AQAFEQDR	98	105	101	1	Method 2	LED
AQAFEQDR	98	105	103-104	1	Method 2	LED
AQAFEQDR	98	105	101	22	Method 2	Hg-Xe
AQAFEQ‡DR	98	105	102	9	Method 2	Hg-Xe
AQAFEQDR	98	105	102	1	Method 2	Hg-Xe
AQAFEQDR	98	105	103	1	Method 2	Hg-Xe
GSHMADKIAIVNMGSLFQQVAQK	17	39	27-29	1	Method 3	LED
IAIVNMGSLFQQVAQK	24	39	27	5	Method 3	LED
IAIVNMGSLFQQVAQK	24	39	27-29	8	Method 3	LED
IAIVNMGSLFQQVAQK	24	39	34	1	Method 3	LED
TGVSNTLENEFKGR	40	53	40-42	1	Method 3	LED
TGVSNTLENEFKGR	40	53	42	3	Method 3	LED
TGVSNTLENEFKGR	40	53	42-44	4	Method 3	LED
TGVSNTLENEFKGR	40	53	42-45	1	Method 3	LED
TGVSNTLENEFKGR	40	53	46	1	Method 3	LED
TGVSNTLENEFKGR	40	53	47	17	Method 3	LED
TGVSNTLENEFK	40	51	47	24	Method 3	LED
T†GVSNTLENEFK	40	51	47	9	Method 3	LED
TGVSNTLENEFKG	40	52	47	9	Method 3	LED
TGVSNTLENEFKGR	40	53	51	11	Method 3	LED
TGVSNTLENEFKGR	40	53	43	7	Method 3	LED
TGVSNTLENEFKGR	40	53	44	1	Method 3	Hg-Xe

TGVSNTLENEFK	40	51	47	24	Method 3	Hg-Xe
TGVSNTLENEFKGR	40	53	47	18	Method 3	Hg-Xe
TGVSNTLENEFKGR	40	53	51	12	Method 3	Hg-Xe
ASELQR	54	59	54	2	Method 3	Hg-Xe
ASELQR	54	59	56	2	Method 3	Hg-Xe
ASELQRMETDLQAK	54	67	60	8	Method 3	Hg-Xe
ASELQRMETDLQAK	54	67	59-61	5	Method 3	Hg-Xe
RM*ETDLQAK	59	67	59-60	10	Method 3	Hg-Xe
M†ETDLQAK	60	67	60	23	Method 3	LED
M*ETDLQAK	60	67	60-61	8	Method 3	LED
METDLQAK	60	67	60-61	4	Method 3	LED
M*ETDLQAK	60	67	60	40	Method 3	Hg-Xe
METDLQAK	60	67	60	16	Method 3	Hg-Xe
METDLQAK	60	67	60-61	1	Method 3	Hg-Xe
METDLQ‡AK	60	67	63	2	Method 3	Hg-Xe
METDLQ‡AK	60	67	64	1	Method 3	Hg-Xe
AGSDRTKLEKDVMAQR	76	91	83	3	Method 3	LED
AGSDRTKLEKDVMAQR	76	91	83-85	1	Method 3	LED
AGSDRTKLEKDVMAQR	76	91	83	3	Method 3	Hg-Xe
AGSDRTKLEKDVMAQR	76	91	84	3	Method 3	Hg-Xe
T†KLEKDVMAQR	81	91	81-83	2	Method 3	LED
T†KLEKDVMAQR	81	91	81-84	1	Method 3	LED
TKLEK†DVMAQR	81	91	81-85	1	Method 3	LED
TKLEKDVMAQR	81	91	83	2	Method 3	Hg-Xe
TKLEKDVMAQR	81	91	84	4	Method 3	Hg-Xe
TKLEKDVMAQR	81	91	85	7	Method 3	Hg-Xe
TKLEKDVMAQRQTFAQK	81	97	90-94	13	Method 3	Hg-Xe
LEKDVMAQR	83	91	83	18	Method 3	Hg-Xe
LEKDVMAQ‡R	83	91	83	7	Method 3	Hg-Xe
LEKDVMAQR	83	91	85	1	Method 3	Hg-Xe
DVMAQRQTFAQK	86	97	90-95	1	Method 3	Hg-Xe
DVMAQRQTFAQK	86	97	90-94	4	Method 3	Hg-Xe
QTFAQKAQAFEQDR	92	105	97	4	Method 3	LED
QTFAQKAQAFEQDR	92	105	102	5	Method 3	LED
QTFAQKAQAFEQDRAR	92	107	97	2	Method 3	Hg-Xe
QTFAQKAQAFEQDR	92	105	97	7	Method 3	Hg-Xe
AQAFEQDR	98	105	101	27	Method 3	LED
A†QAFEQDR	98	105	102	8	Method 3	LED
AQAFEQDR	98	105	103	3	Method 3	LED
AQAFEQDR	98	105	101	26	Method 3	Hg-Xe
AQAFEQDR	98	105	103	5	Method 3	Hg-Xe
SVANSQDIDLVVDANAVAYNSSDVKDITADVLK	126	158	135-138	6	Method 3	Hg-Xe
SVANSQDIDLVVDANAVAYNSSDVKDITADVLK	126	158	139	1	Method 3	Hg-Xe
**W7C**						
M*ETDLQAK	60	67	60-61	1	Method 1	LED
M*ETDLQAK	60	67	60	1	Method 1	LED
METDLQAK	60	67	61	1	Method 1	LED
M*ETDLQAK	60	67	60-61	1	Method 1	Hg-Xe
METDLQAK	60	67	61	1	Method 1	Hg-Xe
AQAFEQDR	98	105	102	2	Method 1	LED
AQAFEQDR	98	105	101	1	Method 1	LED

AQAF**E**QDR	98	105	102	1	Method 1	Hg-Xe
**TGV**SNTLENEFKGR	40	53	40-42	1	Method 3	LED
TGV**S**NTLENEFKGR	40	53	43	7	Method 3	LED
TGVSNTL**E**NEFKGR	40	53	47	7	Method 3	LED
TGVSNTL**E**NEFK	40	51	47	13	Method 3	LED
TGVSNTLEN**EFKGR**	40	53	49-53	1	Method 3	LED
TGVSNTLENEF**K**GR	40	53	51	7	Method 3	LED
**RM***ETDLQAK	59	67	59-60	10	Method 3	LED
**RM*****E**TDLQAK	59	67	59-61	1	Method 3	LED
**M***ETDLQAK	60	67	60	18	Method 3	LED
**M**ETDLQAK	60	67	60	8	Method 3	LED
M***E**TDLQAK	60	67	61	1	Method 3	LED
**L**EKDVMAQR	83	91	83	1	Method 3	LED
AQA**F**EQDR	98	105	101	23	Method 3	LED
AQAF**E**QDR	98	105	102	1	Method 3	LED
AQAFE**Q**DR	98	105	103	4	Method 3	LED
SVA**NSQ‡DIDLVVD**ANAVAYNSSDVKDITADVLK	126	158	130-138	1	Method 3	LED
SVANSQDIDLVV**D**ANAVAYNSSDVKDITADVLK	126	158	138	2	Method 3	LED

Table S5: Skp peptides identified with modifications from MTS-TFMD-conjugated T144C and W7C OmpA. * = oxidation, † = carbamylation, ‡ = deamidation.

Sequence	Start Residue	End Residue	Modified Residue	Number of Spectra	Enrichment	Lamp
**T144C**						
GSHMADKIAIVNMGSLFQQVAQK	17	39	29-32	5	Method 3	LED
GSHMADKIAIVNMGSLFQQVAQK	17	39	33	1	Method 3	LED
GSHMADKIAIVNMGSLFQQVAQK	17	39	34	1	Method 3	LED
TGVSNTLENEFKGR	40	53	40-41	2	Method 3	LED
TGVSNTLENEFKGR	40	53	42-43	14	Method 3	LED
TGVSNTLENEFK	40	51	42	1	Method 3	LED
TGVSNTLENEFKGR	40	53	43	45	Method 3	LED
TGVSNTLENEFK	40	51	43	46	Method 3	LED
T†GVSNTLENEFKGR	40	53	43	1	Method 3	LED
TGVSNTLENEFKGR	40	53	42-44	1	Method 3	LED
TGVSNTLENEFKGR	40	53	45	9	Method 3	LED
TGVSNTLENEFKGR	40	53	46	4	Method 3	LED
TGVSNTLENEFKGR	40	53	51	2	Method 3	LED
ASELQR	54	59	58	1	Method 3	LED
METDLQAK	60	67	62	1	Method 3	LED
METDLQAK	60	67	64	2	Method 3	LED
METDLQAK	60	67	65	10	Method 3	LED
TKLEKDVMAQR	81	91	88	15	Method 3	LED
TKLEKDVMAQRQTFAQK	81	97	90-94	38	Method 3	LED
LEKDVMAQRQTFAQK	83	97	90-94	5	Method 3	LED
DVMAQRQTFAQK	86	97	90-94	12	Method 3	LED
AQAFEQDR	98	105	99	6	Method 3	LED
AQAFEQDR	98	105	100	1	Method 3	LED
AQAFEQDRAR	98	107	101-102	5	Method 3	LED
AQAFEQDR	98	105	101	3	Method 3	LED
AQAFEQDRAR	98	107	102	2	Method 3	LED
AQAFEQDR	98	105	102	3	Method 3	LED
AQAFEQDRAR	98	107	105-107	25	Method 3	LED
AQAFEQDRAR	98	107	106-107	14	Method 3	LED
RSNEERGKLVTR	108	119	113	12	Method 3	LED
SNEERGKLVTR	109	119	113	7	Method 3	LED
GKLVTR	114	119	116	8	Method 3	LED
**W7C**						
TGVSNTLENEFKGR	40	53	42	27	Method 3	LED
TGVSNTLENEFK	40	51	42	31	Method 3	LED
TGVSNTLENEFKGR	40	53	43	62	Method 3	LED
TGVSNTLENEFK	40	51	43	65	Method 3	LED
T†GVSNTLENEFKGR	40	53	43	4	Method 3	LED
TGVSNTLENEFKGR	40	53	45	2	Method 3	LED
TGVSNTLENEFKGR	40	53	46	9	Method 3	LED
TGVSNTLENEFKGR	40	53	51	1	Method 3	LED
ASELQR	54	59	58	4	Method 3	LED
METDLQAK	60	67	60-61	1	Method 3	LED
METDLQAK	60	67	65	2	Method 3	LED
TKLEKDVMAQR	81	91	88	1	Method 3	LED

**AQ**AFEQDR	98	105	98-99	4	Method 3	LED
A**Q**AFEQDR	98	105	99	1	Method 3	LED
AQ**A**FEQDR	98	105	100	6	Method 3	LED
AQA**F**EQDR	98	105	101	8	Method 3	LED
AQAFE**QDRAR**	98	108	103-107	4	Method 3	LED
AQAFEQD**RAR**	98	108	105-107	7	Method 3	LED
AQAFEQDR**A**R	98	108	106	22	Method 3	LED
R**S**NEER	108	113	109	2	Method 3	LED
RSNEE**R**GKLVTR	108	119	113	4	Method 3	LED
SNE**ER**GKLVTR	109	119	112-113	7	Method 3	LED
SNEE**R**GKLVTR	109	119	113	2	Method 3	LED

S4. Supporting References

[1] J. A. Miles, D. J. Yeo, P. Rowell, S. Rodriguez-Marin, C. M. Pask, S. L. Warriner, T. A. Edwards, A. J. Wilson, *Chem Sci* **2016**, *7*, 3694-3702.

[2] B. Schiffrin, A. N. Calabrese, A. J. Higgins, J. R. Humes, A. E. Ashcroft, A. C. Kalli, D. J. Brockwell, S. E. Radford, *J Mol Biol* **2017**, *429*, 3776-3792.

[3] A. Liga, J. A. S. Morton, M. Kersaudy-Kerhoas, *Microfluid Nanofluid* **2016**, *20*, 164.

[4] a)J. R. Hill, A. A. B. Robertson, *J Med Chem* **2018**; b)G. W. Preston, A. J. Wilson, *Chem Soc Rev* **2013**, *42*, 3289-3301.

[5] Q. Liu, T. Moldoveanu, T. Sprules, E. Matta-Camacho, N. Mansur-Azzam, K. Gehring, *J Biol Chem* **2010**, *285*, 19615-19624.

[6] a)B. M. Burmann, C. Wang, S. Hiller, *Nat Struct Mol Biol* **2013**, *20*, 1265-1272; b)B. Schiffrin, A. N. Calabrese, P. W. A. Devine, S. A. Harris, A. E. Ashcroft, D. J. Brockwell, S. E. Radford, *Nat Struct Mol Biol* **2016**, *23*, 786-793; c)T. A. Walton, M. C. Sousa, *Mol Cell* **2004**, *15*, 367-374.

S5. Analytical data for peptides

BID$_{80\text{-}102}$(I83C)-MTS diazirine

Calculated mass: 2840.3462 Da   Observed mass: 2840.3537 Da

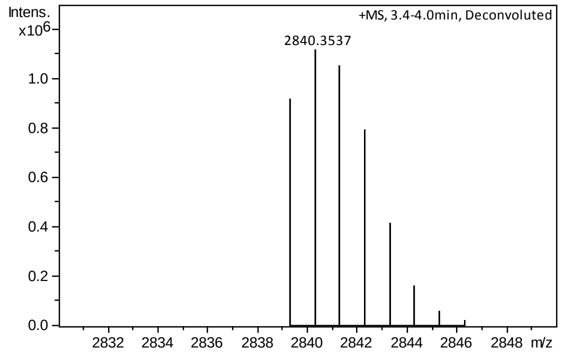

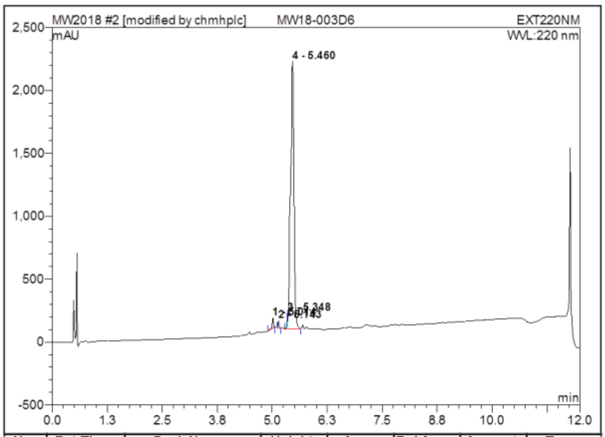

No.	Ret.Time	Peak Name	Height	Area	Rel.Area	Amount	Type
	min		mAU	mAU*min	%		
1	5.01	n.a.	81.045	2.892	1.45	n.a.	BMB
2	5.14	n.a.	56.201	1.755	0.88	n.a.	BMB
3	5.35	n.a.	124.362	3.512	1.76	n.a.	BM
4	5.46	n.a.	2124.613	191.402	95.91	n.a.	MB
Total:			2386.220	199.560	100.00	0.000	

BID$_{80\text{-}102}$(I86C)-MTS diazirine

Calculated mass: 2840.3462 Da   Observed mass: 2840.3559 Da

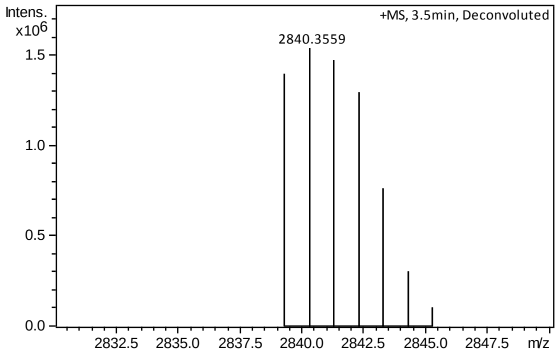

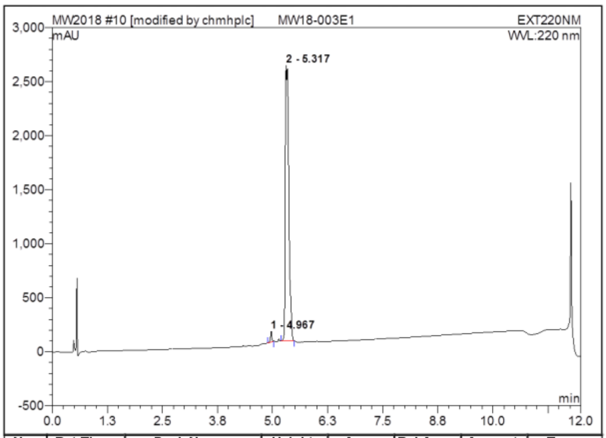

No.	Ret.Time	Peak Name	Height	Area	Rel.Area	Amount	Type
	min		mAU	mAU*min	%		
1	4.97	n.a.	98.063	3.230	1.33	n.a.	BMB
2	5.32	n.a.	2552.472	239.741	98.67	n.a.	BMB*
Total:			2650.535	242.972	100.00	0.000	

BID$_{80\text{-}102}$(V93C)-MTS diazirine

Calculated mass: 2854.3619 Da   Observed mass: 2854.3724 Da

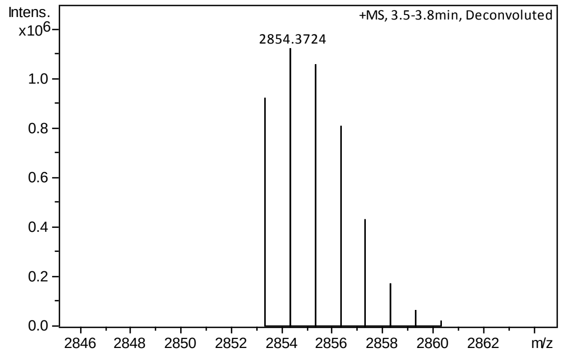

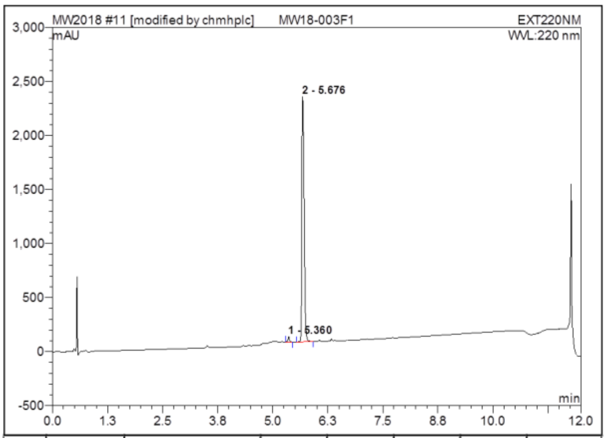

No.	Ret.Time	Peak Name	Height	Area	Rel.Area	Amount	Type
	min		mAU	mAU*min	%		
1	5.36	n.a.	50.073	1.795	1.31	n.a.	BMB
2	5.68	n.a.	2271.900	135.187	98.69	n.a.	BMB
Total:			2321.973	136.982	100.00	0.000	

BID$_{80-102}$(M97C)-MTS diazirine

Calculated mass: 2822.3898 Da Observed mass: 2822.4003 Da

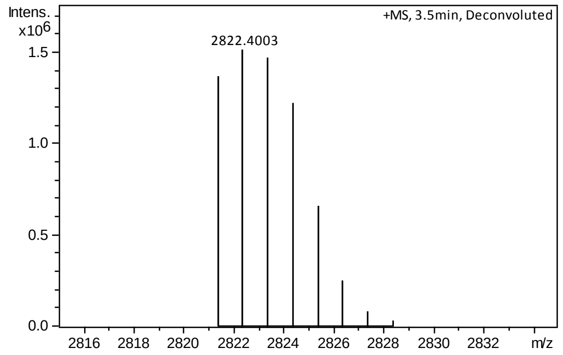

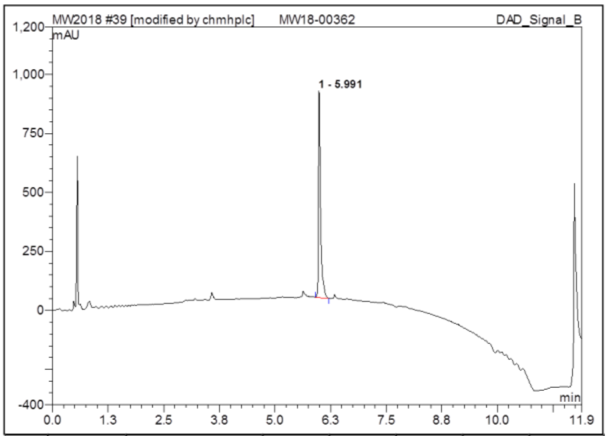

No.	Ret.Time	Peak Name	Height	Area	Rel.Area	Amount	Type
	min		mAU	mAU*min	%		
1	5.99	n.a.	876.959	47.870	100.00	n.a.	BMB
Total:			876.959	47.870	100.00	0.000	

BID$_{80\text{-}102}$(S100C)-MTS diazirine

Calculated mass: 2866.3983 Da   Observed mass: 2866.4056 Da

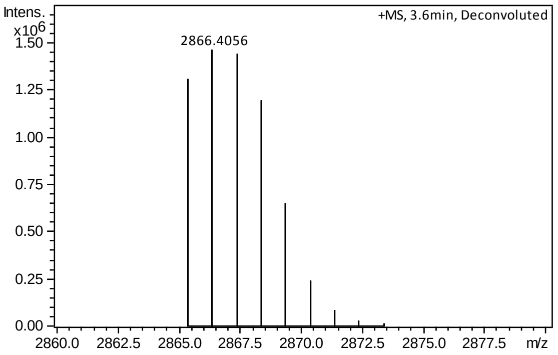

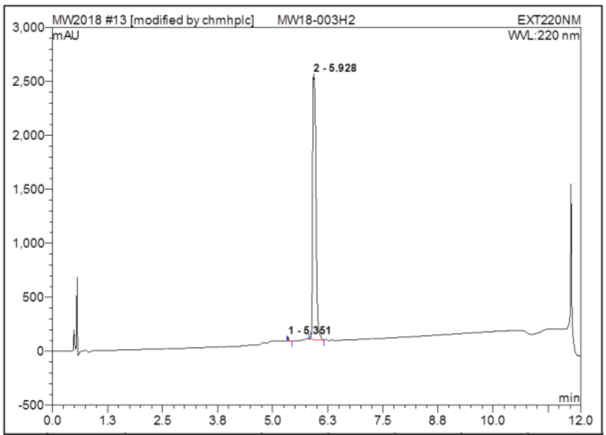

No.	Ret.Time	Peak Name	Height	Area	Rel.Area	Amount	Type
	min		mAU	mAU*min	%		
1	5.35	n.a.	40.800	1.292	0.59	n.a.	BMB
2	5.93	n.a.	2459.946	216.067	99.41	n.a.	BMB*
Total:			2500.747	217.359	100.00	0.000	

BID$_{80\text{-}102}$(I83C)-MTS TFMD

Calculated mass: 2956.3336 Da Observed mass: 2956.2979 Da

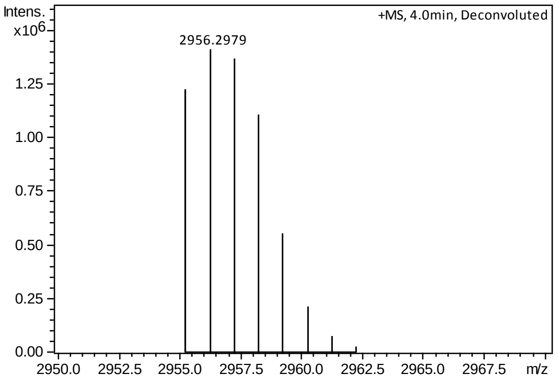

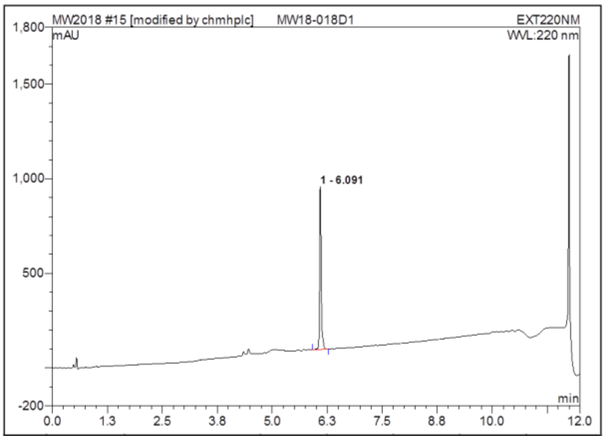

No.	Ret.Time	Peak Name	Height	Area	Rel.Area	Amount	Type
	min		mAU	mAU*min	%		
1	6.09	n.a.	860.458	35.007	100.00	n.a.	BMB
Total:			860.458	35.007	100.00	0.000	

BID$_{80\text{-}102}$(I86C)-MTS TFMD

Calculated mass: 2956.3336 Da   Observed mass: 2956.2905 Da

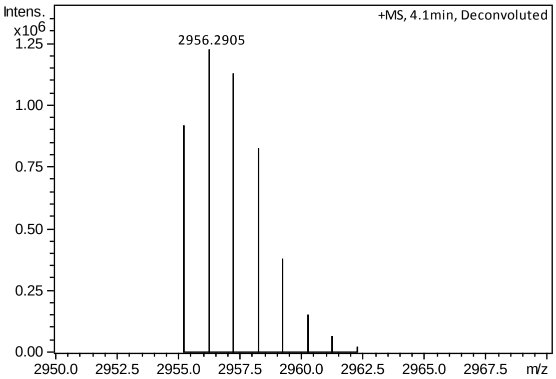

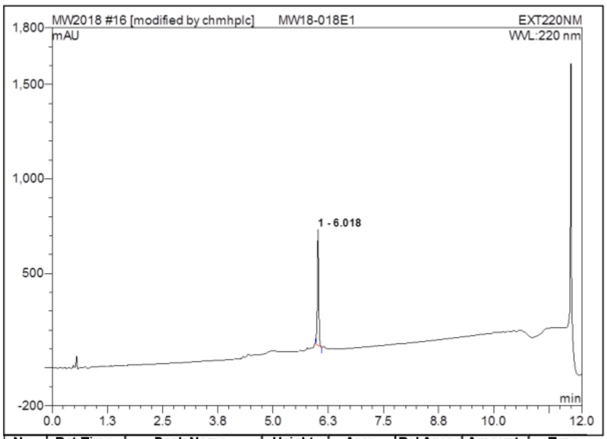

No.	Ret.Time	Peak Name	Height	Area	Rel.Area	Amount	Type
	min		mAU	mAU*min	%		
1	6.02	n.a.	614.403	21.479	100.00	n.a.	BMB
Total:			614.403	21.479	100.00	0.000	

BID$_{80-102}$(V93C)-MTS TFMD

Calculated mass: 2970.3492 Da   Observed mass: 2970.3076 Da

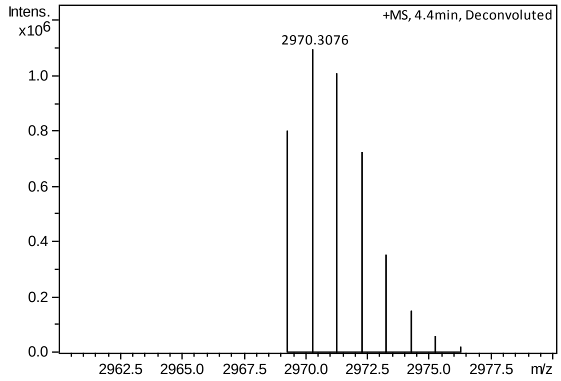

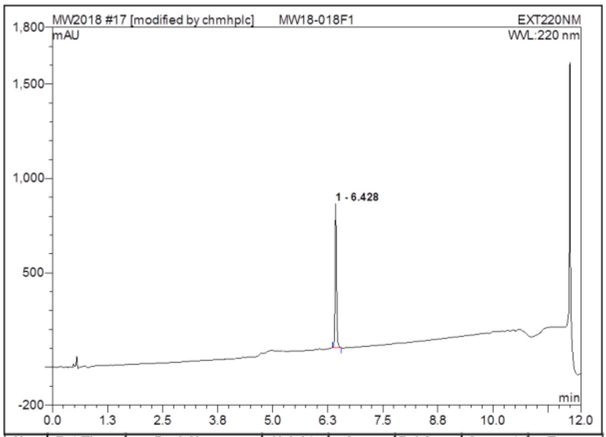

No.	Ret.Time	Peak Name	Height	Area	Rel.Area	Amount	Type
	min		mAU	mAU*min	%		
1	6.43	n.a.	763.823	30.050	100.00	n.a.	BMB
Total:			763.823	30.050	100.00	0.000	

BID$_{80-102}$(M97C)-MTS TFMD

Calculated mass: 2938.3772 Da   Observed mass: 2938.3356 Da

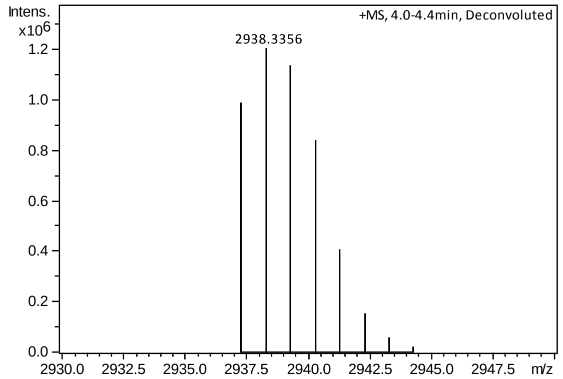

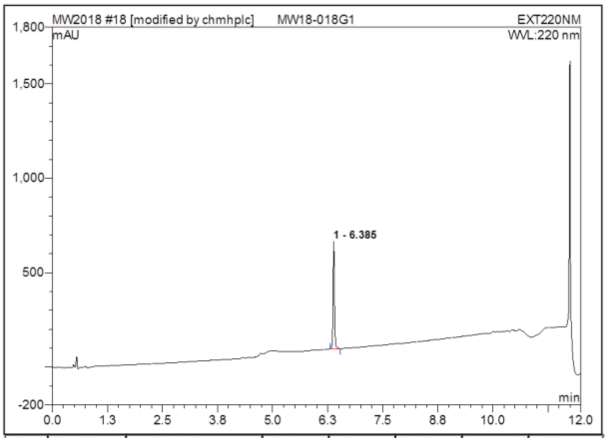

No.	Ret.Time	Peak Name	Height	Area	Rel.Area	Amount	Type
	min		mAU	mAU*min	%		
1	6.39	n.a.	569.706	21.214	100.00	n.a.	BMB
Total:			569.706	21.214	100.00	0.000	

BID$_{80\text{-}102}$(S100C)-MTS TFMD

Calculated mass: 2982.3856 Da   Observed mass: 2982.3207 Da

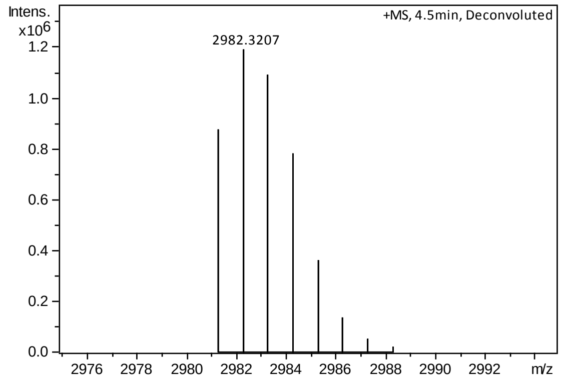

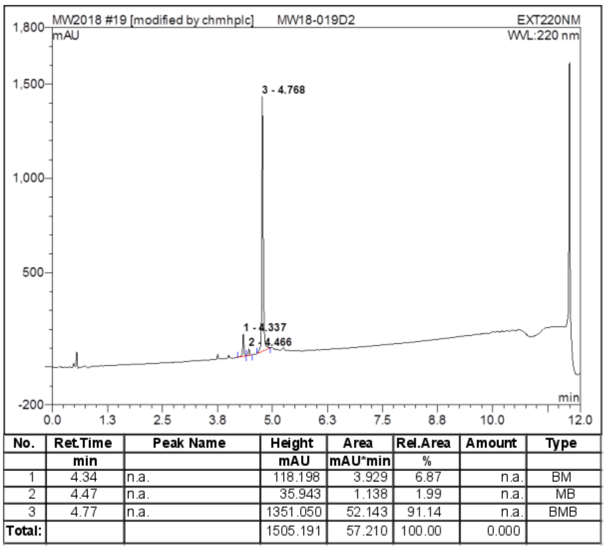

No.	Ret.Time	Peak Name	Height	Area	Rel.Area	Amount	Type
	min		mAU	mAU*min	%		
1	4.34	n.a.	118.198	3.929	6.87	n.a.	BM
2	4.47	n.a.	35.943	1.138	1.99	n.a.	MB
3	4.77	n.a.	1351.050	52.143	91.14	n.a.	BMB
Total:			1505.191	57.210	100.00	0.000	
